# Comparing training window selection methods for prediction in non‐stationary time series

**DOI:** 10.1111/bmsp.70018

**Published:** 2026-01-13

**Authors:** Fridtjof Petersen, Jonas M. B. Haslbeck, Jorge N. Tendeiro, Anna M. Langener, Martien J. H. Kas, Dimitris Rizopoulos, Laura F. Bringmann

**Affiliations:** ^1^ Department of Psychometrics and Statistics, Faculty of Behavioural and Social Sciences University of Groningen Groningen The Netherlands; ^2^ Psychological Methods Group University of Amsterdam Amsterdam The Netherlands; ^3^ Department of Clinical Psychological Science University of Maastricht Maastricht The Netherlands; ^4^ Graduate School of Advanced Science and Engineering Hiroshima University Higashihiroshima Japan; ^5^ Department of Biomedical Data Science Center for Technology and Behavioral Health, Geisel School of Medicine at Dartmouth Lebanon New Hampshire USA; ^6^ Groningen Institute for Evolutionary Life Sciences University of Groningen Groningen The Netherlands; ^7^ Department of Biostatistics Erasmus University Medical Center Rotterdam The Netherlands; ^8^ Department of Epidemiology Erasmus University Medical Center Rotterdam The Netherlands

**Keywords:** dynamic prediction, ecological momentary assessment (EMA), intensive longitudinal data, non‐stationarity, passive sensing

## Abstract

The widespread adoption of smartphones creates the possibility to passively monitor everyday behaviour via sensors. Sensor data have been linked to moment‐to‐moment psychological symptoms and mood of individuals and thus could alleviate the burden associated with repeated measurement of symptoms. Additionally, psychological care could be improved by predicting moments of high psychopathology and providing immediate interventions. Current research assumes that the relationship between sensor data and psychological symptoms is constant over time – or changes with a fixed rate: Models are trained on all past data or on a fixed window, without comparing different window sizes with each other. This is problematic as choosing the wrong training window can negatively impact prediction accuracy, especially if the underlying rate of change is varying. As a potential solution we compare different methodologies for choosing the correct window size ranging from frequent practice based on heuristics to super learning approaches. In a simulation study, we vary the rate of change in the underlying relationship form over time. We show that even computing a simple average across different windows can help reduce the prediction error rather than selecting a single best window for both simulated and real world data.

## 1 INTRODUCTION

Intensive longitudinal data collection has expanded rapidly, often using experience sampling methods (ESM) and passive sensing (Carey et al., 2020). ESM involves participants repeatedly completing questionnaires to capture dynamic behaviours, emotions and contexts (Shiffman et al., 2008), while passive sensing collects smartphone sensor data, such as location data via GPS (Jacobson & Chung, 2020; Torous et al., 2016). This research, especially in psychology, focuses on using passive sensing to build models that predict symptoms or emotions, such as positive and negative affect, obtained via ESM, aiming to improve mental health screening, monitor symptom progression and enable early interventions (Balliu et al., 2024; Langener, Stulp, et al., 2024; Spinazze et al., 2019). For example, predicting binge‐drinking episodes ahead of time could allow for timely alternative behavioural interventions. However, a major obstacle is that models become unstable, and their predictive performance degrades over time (Riley & Collins, 2023).

This degradation in model performance is to be expected given what we know about the development of psychological symptoms in general: Symptoms and their relationships with each other often change in both abrupt and gradual ways over time (Albers & Bringmann, 2020). This phenomenon, known as non‐stationarity (for an introduction in psychology, see Ryan et al., 2023), likely extends to relationships between symptoms and passive data. While various models have been developed to explain and describe non‐stationarity in psychological symptoms (Albers & Bringmann, 2020; Bringmann et al., 2017; Haslbeck et al., 2021), prediction in the context of monitoring and mental health screening has different model requirements: Not only is our main concern high predictability rather than explanation, but we additionally want to be able to adapt to new data coming in so we can use them to intervene in time. Yet, limited attention has been given to how to train prediction models most efficiently while accounting for non‐stationarity during the training process. Thus, this paper addresses this gap by exploring prediction methods that can handle both non‐stationarity and real‐time data.

**FIGURE 1:**
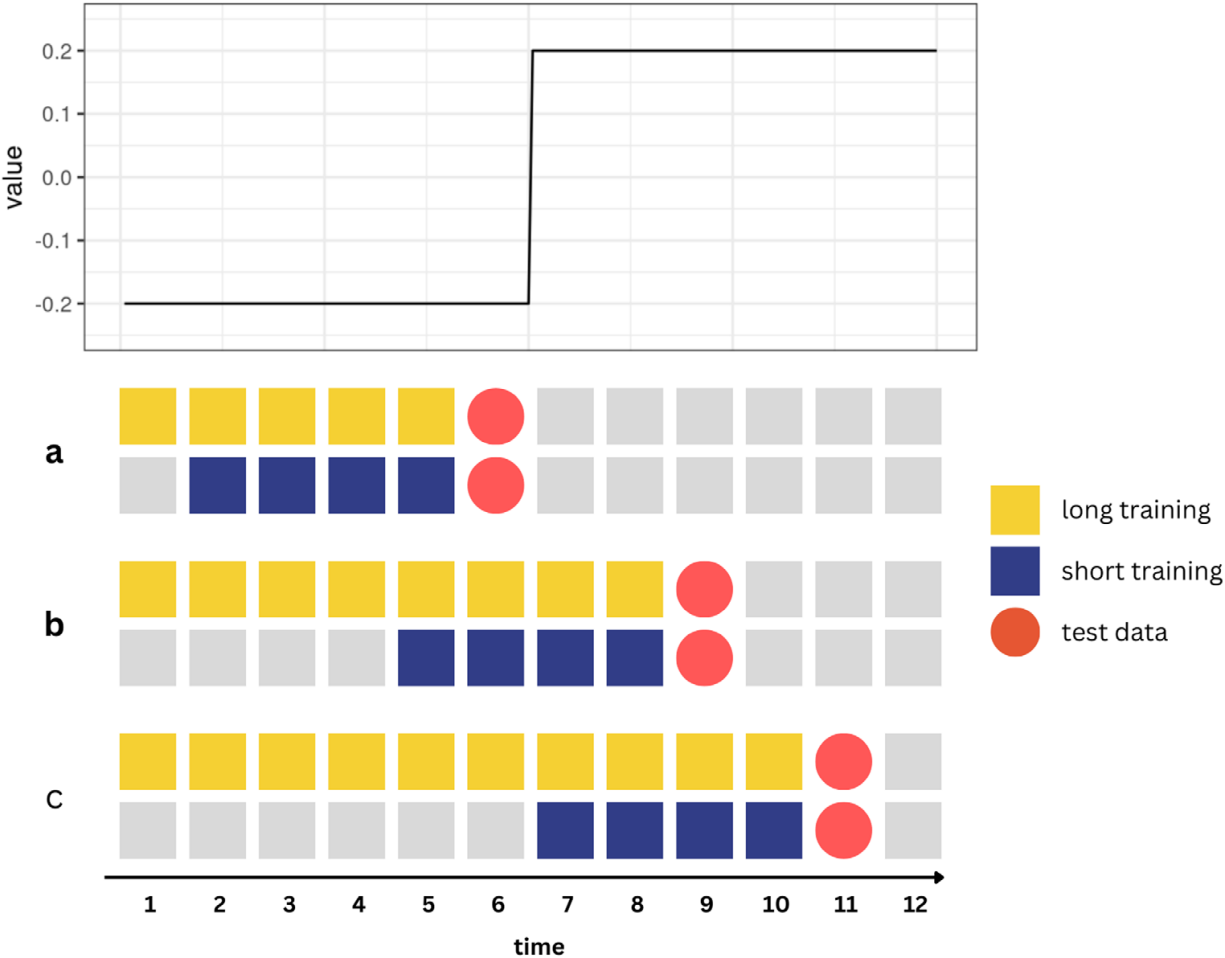
Example of fixed training window approach for performing one‐step‐ahead predictions. The top panel shows an underlying shift in the coefficient at time 6 (from ‐0.2 to 0.2). In scenario (a), the long (yellow) and short (blue) training windows both contain only information before the shift. In scenario (b), the long window includes more old information, whereas the short window contains an equal amount of old and new information. In (c), the short window contains only data from after the shift, and fully captures the new coefficient value. In contrast, the long window still incorporates old information from before the shift.

A common approach to time series prediction is to refit the model each time new data become available, thereby updating predictions based on the latest information (Jacobson & Chung, 2020; Jacobson & Feng, 2022; Langener, Bringmann, et al., 2024; Langener, Stulp, et al., 2024). However, if underlying relationships change, using all past data may lead to inaccurate predictions. For instance, if the optimal model in Week 4 differs greatly from Week 1, data from Week 1 should not inform Week 4 predictions. One solution is the ‘training window’ approach, where the model is refitted only on more recent observations. By excluding older, less relevant data, we can better account for changes in underlying relationships (see Figure 1). The training window approach assumes that using only a subset of past data (rather than all available data) will obtain the most accurate predictions; however, the choice of the window should align with theoretical and empirical observations. Consider an example where someone transitions from work to vacation: The relationship between mood and passively detected social interactions might shift from negative (due to stressful interactions with work colleagues) to positive (from relaxing time with friends). A window focused on the vacation period may yield more accurate predictions than one spanning both contexts, as it captures a more stable pattern that occurs during the vacation period. However, this approach cannot account for non‐stationarity within the window itself, such as mood shifts due to conflicts during the vacation. Regardless, training windows are a practical and often used approach for handling non‐stationarity in prediction settings (Gama et al., 2014; Verachtert et al., 2022).

The training window size determines the rate of ‘forgetting’ old data, and should ideally match the rate of change in the underlying non‐stationarity. Yet, researchers often fix the window size across all participants, assuming the same rate of change for everyone. For example, Jacobson and Feng (2022) used a 24‐h moving window for every individual. However, given individual differences in psychological symptom dynamics, it is implausible that everyone's system changes at the same rate. In fact, initial research suggests that the optimal window size varies across samples (Halder et al., 2017; Kathan et al., 2022; Wang et al., 2016) and individuals (Langener, Bringmann, et al., 2024; Langener, Stulp, et al., 2024).

Our study is the first to use a simulation‐based approach to investigate the impact of training window choices on predictive accuracy. While previous studies have evaluated single training windows in empirical datasets (Langener, Bringmann, et al., 2024; Langener, Stulp, et al., 2024; Verachtert et al., 2022; Wang et al., 2016), no study has systematically explored how to optimally select or combine models with varying training windows in a simulation setting. We introduce and compare commonly used methods for determining the appropriate training window size, as well as more complex new methods which allow us to consider multiple possible window sizes simultaneously. We evaluate these methods in a simulation study, varying the degree of non‐stationarity that can be expected in digital phenotyping studies, and assess their predictive accuracy. Finally, we apply the methods to two real‐world datasets (Langener, Bringmann, et al., 2024), compare the results to the simulation findings and provide R code for replication.

## 2 METHODS

Our approach to training window selection is organized into three parts. First, we describe the time‐varying linear data‐generating process that we consider and the construction of a model library where we fit both ordinary least squares (OLS) and random forest (RF) models with varying training windows to the data (Section 2.1). Next, we present the model selection methods we evaluated, from standard heuristics to advanced approaches like the super learner (Section 3.1‐3.3). Finally, we introduce the forward validation procedure used to assess predictive performance and construct super learners (Section 3.4).

### 2.1 Data‐generating process

The collected sample is denoted as $𝒟=\{(y_{t},x_{t});t=1,\text{…},T\}$, where $y_{t}$ is the dependent variable to be predicted (for example, a psychological variable such as positive affect obtained via ESM) and $x_{t}$ is a vector of p covariates representing features obtained from sensor data. Our goal is to approximate this relationship between the random variable $Y_{t}$ and the random variables $X_{t}$ as closely as possible by fitting a library of models to the data and selecting the procedure that minimizes the error. The true data‐generating process is specified as:

(1) $$Y_{t}=f_{t}(X_{t},\beta_{t}^{*})+\epsilon_{t},$$

where $f_{t}(\cdot )$ determines the true model and defines the relationship between the outcome $Y_{t}$ and the covariate vector $X_{t}$. The function $f_{t}(\cdot )$ can vary over time, as the subscript t indicates. $\beta_{t}^{*}$ denotes the true parameters that describe the true model $f_{t}(\cdot )$. While we focus on a linear relationship, the parameter vector could be of any size to represent any parametric model. The random error $\epsilon_{t}$ represents unmeasured contributions not captured by the covariates and has a normal distribution with mean zero and variance $\sigma^{2}$.

For illustrative purposes, we use a simple linear relationship. As there has been no or limited simulation research on this topic, we chose a linear function because the results and simulation setup are directly interpretable, allowing us to clearly observe how coefficients change over time It takes the form:

(2) $$f_{t}(X_{t},\beta_{t})=X_{t}^{⊤}\beta_{t},$$

where $\beta_{t}$ denotes the vector of regression coefficients at time t. The index t indicates that the coefficients are allowed to vary over time.

**FIGURE 2:**
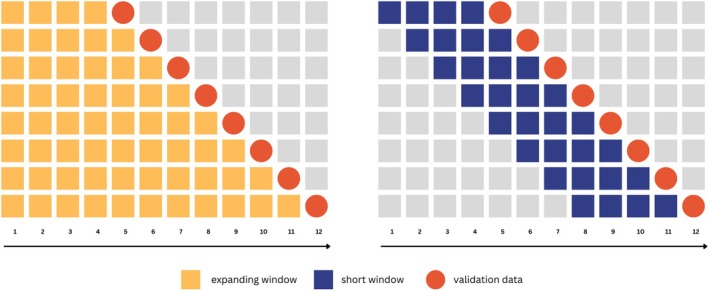
Illustration of one‐step‐ahead predictions (red) for models trained on all past data (yellow) and the last four data points (blue).

Now that the data‐generating mechanism f(·) has been defined, we describe how a model can be fit to approximate the underlying relationship based on the observed values of $y_{t}$ and $x_{t}$. In order to account for differences between individuals in their underlying change, we do not fit a model with a single training window (e.g. the last 24h); rather, we fit a library of candidate models denoted as ${𝒦}_{t}=\{M_{1},\text{…},M_{K}\}$. The included models differ in the size of their training window $w_{k}$. The subscript t denotes the time point at which the models were updated. An example of two models from a potential library can be seen in Figure 2. For example, in the third row of the left panel, we see a model that has been estimated on all time points up to and including t=6, while the model on the right only uses the last four data points to estimate the parameters:

Based on the estimated models, we can make one‐step‐ahead predictions. For model $M_{k}$, we obtain the following:

(3) $${ŷ}_{t+1}^{\left(k\right)}={\hat{f}}_{k}(x_{t+1},{\hat{\beta }}_{kt}|D_{k})\;\text{with}\;D_{k}=\{y_{t-w_{k}+1:t},x_{t-w_{k}+1:t}\}.$$

Here, ${\hat{f}}_{k}(\cdot )$ denotes the estimated model that produces the prediction ${ŷ}_{t+1}^{\left(k\right)}$ based on the covariate vector $x_{t+1}$ and the estimated parameters ${\hat{\beta }}_{kt}$ at time point t. The parameters are estimated given the training data $D_{k}$, where $w_{k}$ denotes the model‐specific training window. For example, the model on the right panel of Figure 2 has a training window of $w_{k}=4$, and as a result, the training data for this model is $D_{k}=\{y_{t-3},y_{t-2},y_{t-1},y_{t};\;x_{t-3},x_{t-2},x_{t-1},x_{t}\}$.

It is also important to mention the different ways the covariates (phenotyping data) can be aggregated to match the time scale of the outcome (ESM data). The sensor data are collected with a high frequency (multiple times per minute), while ESM data are usually only obtained a few times daily. To overcome this temporal mismatch, researchers frequently create summary measures of the sensor data across a specific time interval (e.g. 1, 3, 6 h), resulting in different features such as the mean, standard deviation or maximum. When we refer to our covariates via $x_{t}$, these represent the summarized features aggregated from the raw sensor data.

**FIGURE 3:**
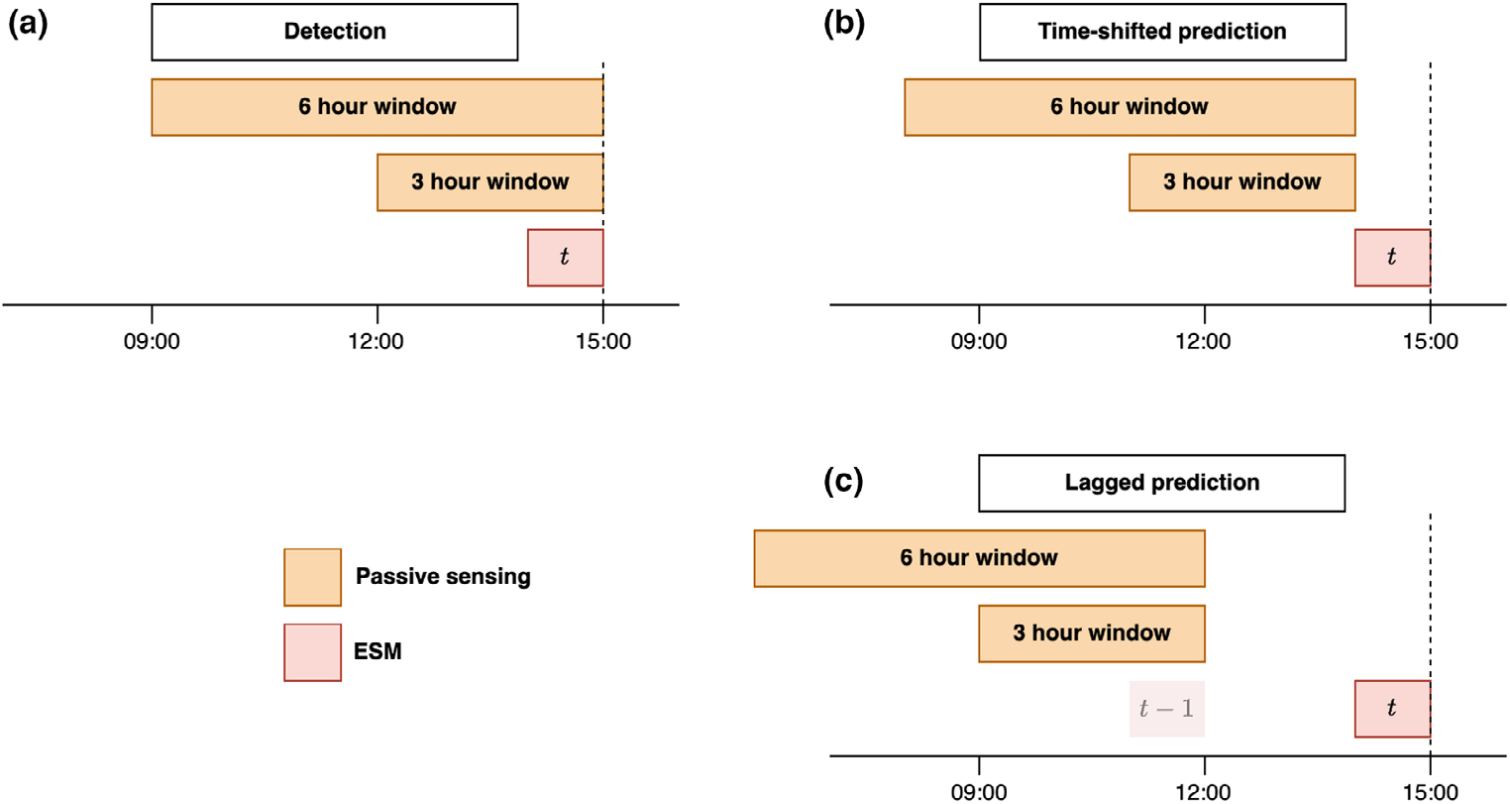
Illustration of different ways that passive sensing data can be matched to the ESM data in terms of timescale. In the case of (a) detection, the time interval of aggregation (in orange) includes the moment when the ESM questionnaire was completed. In the case of (b) time‐shifted prediction, the time interval ends 1 h before the ESM questionnaire. In (c) lagged prediction, the passive data are aggregated up to and including the previous ESM assessment. Note however that the aggregated passive sensing data is linked to the ESM assessment at the current time point, not the previous one.

However, the temporal alignment between passive sensing features and ESM outcomes can be implemented in several different ways, which change the interpretability and possible applications of the prediction models (see Figure 3). In the *detection* approach (Figure 3a), the passive sensing data are aggregated up to and including the exact moment when the ESM questionnaire is completed (Bae et al., 2017, 2018; Langener, Stulp, et al., 2024). In the *time‐shifted* approach, the data is aggregated up to 1 h (for example) before the questionnaire (Figure 3b), creating a temporal gap that can be useful if the goal is to intervene before binge drinking happens, for example (Bae et al., 2023). The *lagged* approach (Figure 3c) aligns with traditional time‐series research in psychology by using passive sensing data summarized up to the previous ESM assessment to predict the current outcome, similar to how previous ESM measurements are usually linked to measurements at the current moment (Bringmann et al., 2013). In the current article, we focus on the *detection*approach as it has been used, for example, in binge drinking detection (Bae et al., 2017, 2018), though the training window selection methods we propose are applicable across all temporal alignment strategies.

## 3 TRAINING WINDOW SELECTION METHODS

In our study, we have the candidate library ${𝒦}_{t}$ of models that differ in their training window size and aim to capture the underlying data‐generating mechanism closely. However, the optimal predictive model (or combination of models) is generally unknown; we need a method to select the model that minimizes the prediction error. We discuss several methods for selecting the optimal model to address this challenge. First, we describe methods that select a single model, and later, methods that simultaneously combine and select different models.

### 3.1 Single model methods

One common method for selecting a training window is the *expanding window* method, denoted as $f_{\exp}(\cdot )$, where all past data up to the current time point is used to train the model (e.g., Balliu et al., 2024). As the training window is set as the current time point $w_{k}=t$, this approach eliminates the need to decide on the specific size of the training window data, and no data are ever discarded (see left panel of Figure 2).

Another approach is to select a specific training window that contains one or more days of data. The exact number of data points within the window depends on the number of ESM signals collected per day. For example, in the last day window method, denoted as $f_{24}(\cdot )$, the training window includes all data points from the past 24 h. In contrast, the last two days window, $f_{48}(\cdot )$, contains data points from the past 48 h. This heuristic‐based method aims to account for potential changes in the underlying relationship over time by discarding data older than the predefined threshold (see, e.g. the right panel of Figure 2). A key difference from the expanding window is that the size of the window needs to be chosen and, in this case, is based on a heuristic rule.

Both approaches, however, assume that the training window (a) accurately captures the change in the underlying data‐generating mechanism and (b) is the same for every individual in the sample. As the rate of change in the mechanism likely differs between people, these approaches can result in poor predictions. Instead of relying on a single fixed window for all individuals, researchers could benefit from comparing a wider range of training windows to find the optimal one for each individual.

### 3.2 Average

A relatively simple way to consider multiple possible windows and to take into account differences across individuals is to compute the average of the predictions from all models, which we denote as ${\hat{f}}_{\text{avg}}$. Averaging predictions across all models mitigates the risk of selecting an inadequate model by considering multiple plausible alternatives (given that the true model is not among the candidate models). It is motivated by findings from studies like the M4 forecasting competition, where simple averages often outperform individual models or more sophisticated combinations of multiple models (Makridakis et al., 2020).

However, simple averaging treats all models the same, regardless of their individual predictive performance, because well‐performing models are weighted equally with poorly performing ones. By assigning greater weights to models with higher predictive accuracy, super learning can improve upon simple averaging.

### 3.3 Super learners

Super learning is a general method to combine different prediction models or algorithms to optimize prediction accuracy for an outcome of interest (Van Der Laan et al., 2007). The combined prediction theoretically performs at least as well as the best individual model. The optimal combination is usually achieved by minimizing the prediction error, such as the mean squared prediction error (MSPE) of all candidate models, which measures predictive performance.

In general, the MSPE for a given model is defined as follows:

(4) $$\text{MSPE}=E\left[{\left\{Y_{t+1}-\hat{f}(X_{t+1},\hat{\beta })\right\}}^{2}\right],$$

where the expectation is taken with respect to the true data‐generating distribution. In (4), we use a general definition of predictions ${Ŷ}_{t+1}=\hat{f}(X_{t+1},\hat{\beta })$, describing the predictive ability of any procedure that obtains predictions. This includes evaluating models in the library ${𝒦}_{t}$, consisting of individual training window models (see Equation 3), as well as procedures like the average or super learner, which are based on multiple individual models.

In practice, we do not have access to the true MSPE value, as the data‐generating distribution $f_{t}(X_{t},\beta_{t}^{*})$ is unknown. Instead, we estimate the error, denoted $\text{M}\hat{\text{SP}}\text{E}$, often using cross‐validation to account for overfitting. The specific cross‐validation procedure used is discussed in Section 3.4. The estimated $\text{M}\hat{\text{SP}}\text{E}$ is then used to construct the super learner by combining models in the candidate library ${𝒦}_{t}$, corresponding to OLS or RF models with different training windows $w_{k}$.

The estimated $\text{M}\hat{\text{SP}}\text{E}$ errors are then used to combine predictions of all candidate models. Each model in ${𝒦}_{t}$ is assigned a weight, with weights summing to one and constrained between zero and one, as the super learner is a weighted average of all models. The weights give more impact to models that predict better. The resulting prediction is denoted as

(5) $${ŷ}_{t+1}={\hat{f}}_{\text{eSL}}(x_{t+1})=\overset{K}{\underset{k=1}{\sum }}{\hat{\omega }}_{k}\hat{f}(x_{t+1},\hat{\theta }|D_{k}),$$

where ${\hat{\omega }}_{k}$ is the weight of each model. This prediction is called an *ensemble super learner* (eSL) as it combines different models. We also consider the *discrete super learner* (dSL), which selects the model with the lowest $\text{M}\hat{\text{SP}}\text{E}$ and is often referred to as the *cross‐validation selector*. We include because it has been suggested to use the model with the training window that has the lowest cross‐validated prediction error (Langener, Stulp, et al., 2024; Verachtert et al., 2022), and it often outperforms the more complicated eSL (Rizopoulos & Taylor, 2024). For more details on implementing both methods, see the ‘Super Learner’ section in the Supplementary Material.

### 3.4 Forward validation

To construct the super learner, we need to estimate the prediction errors of all candidate models. While k‐fold cross‐validation is commonly used, standard methods assume independent data points and are unsuitable for time series data with temporal dependencies. Therefore, we implement a cross‐validation approach that accounts for these dependencies.

Time series cross‐validation addresses this by dividing the dataset into folds consisting of sequential data points, where each fold is larger than the previous one. Each fold is split into a training set (all but the last observation) and a validation set (the last observation). See Figure 2 for an example. The model is trained on the training set and used to make a one‐step‐ahead prediction for the validation set. The prediction error is calculated by comparing predicted and actual observations. Repeating this across all folds yields an estimate of model performance, typically measured using MSPE. By applying this procedure to all models in ${𝒦}_{t}$, we can estimate their prediction errors and construct the super learners based on this estimate.

**FIGURE 4:**
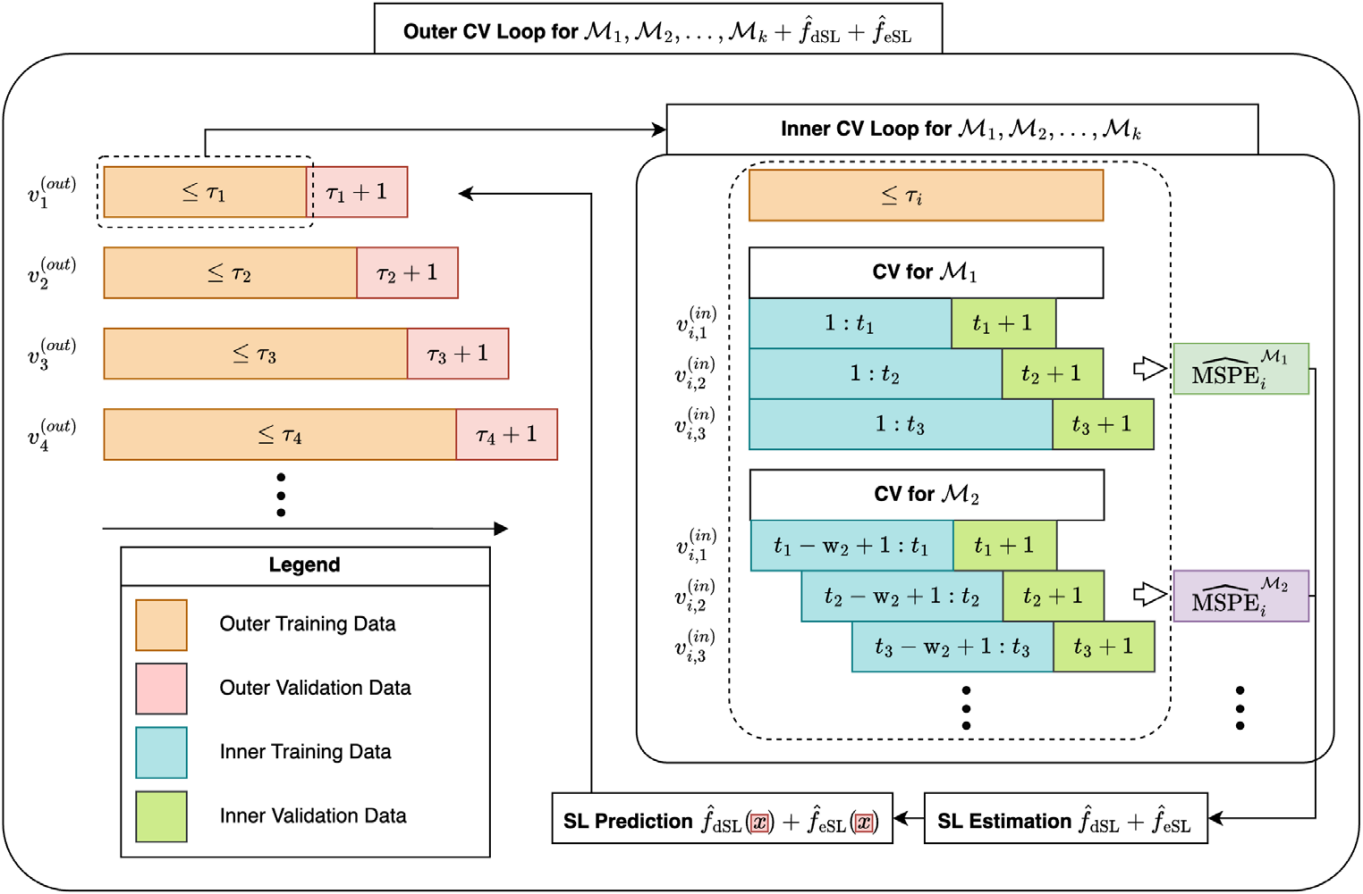
Example of forward validation. The inner loop estimates prediction errors for different candidate models and constructs super learners. The outer loop is used to estimate the prediction error of super learners as well as candidate models. On the right panel, you can see an example of how the first outer fold is split into an internal validation loop.

To evaluate the predictive performance of the super learner itself—not just the candidate models—we introduce the *forward validation* approach (Malenica et al., 2023). This nested time series cross‐validation method consists of an outer and inner loop, where the inner loop is nested within the outer loop, and we perform time series cross‐validation iterations in both loops (see Figure 4 for an illustration). In the outer loop (left side), we evaluate the super learner's predictive accuracy by testing it on validation data (red) not used in its construction. This procedure is similar to that described in Figure 3, except we evaluate the super learner instead of the candidate models. We construct the super learner within each outer loop iteration by performing an additional inner loop on the training data of the outer loop (orange). This means we perform time series cross‐validation again on a smaller dataset to evaluate the error of the candidate models that differ in training window size, as indicated by the blue inner training data. In the example on the right side of Figure 4, we evaluate the predictive accuracy of model ${ℳ}_{1}$ with an extended training window that grows over time, and a second model ${ℳ}_{2}$ that has a fixed training window of size w. As each outer loop fold grows in size and we repeat the inner loop each time, we can reconstruct the super learner for every new data point that we collect and adapt to changes in the predictive ability of the candidate learners. The detailed procedure is explained in the Supplementary Material.

From a single inner loop, we obtain estimated prediction errors of all candidate models, allowing us to construct both the dSL and eSL as described earlier and retrain all candidate models on the entire outer training data. This leaves us with a complete model library denoted by ${𝒞}_{t}=\{M_{1},\text{…},M_{k},{\hat{f}}_{\text{avg}},{\hat{f}}_{\text{dSL}},{\hat{f}}_{\text{eSL}}\}$ of size K+3. Note that we explicitly include the average selection method in the library, as it also combines different training windows of ${𝒦}_{t}$. The last day $\left(f_{24}\right)$, last 2 days ($f_{48}$) and expanding window selection methods ($f_{\exp}$) are part of ${𝒦}_{t}$, representing fixed training window choices.

Using the entire model library ${𝒞}_{t}$, we can predict the outer validation point at $\tau_{i}+1$ and calculate the prediction error for all models, including the super learner. By averaging prediction errors across all outer fold validation sets, we obtain an estimate of the out‐of‐sample prediction error ${\text{M}\hat{\text{SP}}\text{E}}_{\text{(out)}}^{C}$ for each model in the complete library ${𝒞}_{t}$, allowing us to compare whether the super learners perform better than any given candidate model.

## 4 EVALUATING METHODS USING A SIMULATION STUDY

We set up a simulation study with three main goals: (a) to illustrate how default choices of training windows (like using the last 24 h or an expanding window) can undermine predictive performance when chosen carelessly, (b) to demonstrate how methods like super learners can adaptively select the optimal window size to enhance predictive performance dynamically and individually and (c) to evaluate whether these findings generalize across both linear (OLS) and non‐linear (RF) models, with the latter being commonly used in digital phenotyping research (Benoit et al., 2020).

### 4.1 Setup

Our simulation is designed to match typical digital phenotyping prediction studies such as those by Jacobson and Feng (2022).

#### 4.1.1 Data‐generating OLS model

The underlying true model is a time‐varying linear regression model as discussed before:

(6) $$Y_{t}=f_{t}(X_{t})=X_{t}^{⊤}\beta_{t}+\epsilon_{t}.$$

**FIGURE 5:**
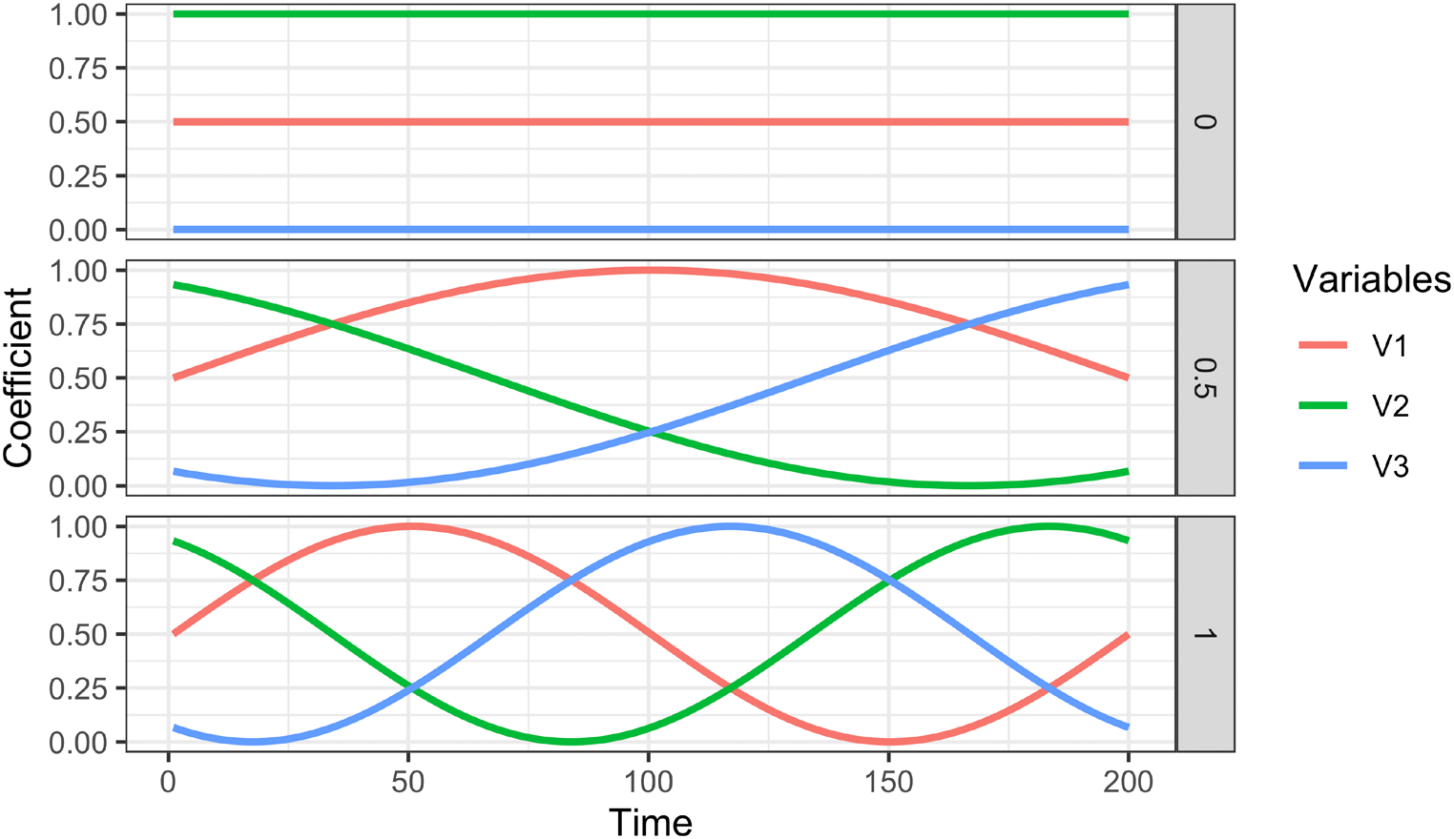
Depiction of different rates of change for the time‐varying coefficients. The first scenario shows no change (rate = 0), the second scenario shows half of an oscillation over the entirety of the time series (rate = 0.5) and the last scenario shows a full oscillation (rate =1).

This model is often referred to as a contemporaneous or Lag‐0 model, as the outcome is related to the covariates at the same time point, rather than lagged covariates. We consider four covariates, allowing us to fit standard OLS as well as RF models for all sample sizes and training windows. We evaluate the results of the OLS and RF models separately. The coefficients $\beta_{t}$ change over time following a sine function. The rate of change of the sine pattern determines how quickly the sine waves vary. We chose a sine function for modeling time‐varying coefficients for several reasons. First, sine functions are frequently used in psychological time series simulations (Bringmann et al., 2017; Haqiqatkhah & Hamaker, 2025) as psychological symptoms and their relationships might exhibit natural cyclical patterns (e.g. daily mood fluctuations, weekly behavioural routines) or smooth temporal transitions. Secondly, digital phenotyping research has investigated whether features follow sinusoidal patterns, and how properties of those waves (amplitude, frequency, phase) relate to mental‐health outcomes (Gao et al., 2023; Zhang et al., 2024). Lastly, and important for our study, sine waves let us directly control how quickly the coefficients change over time. Rather than varying the number of abrupt jumps at specific time points, we simply vary how quickly (or slowly) the sine wave oscillates over time. This gives us more control to examine how different rates of gradual change impact the performance of window selection methods across a continuous spectrum—from no change (rate = 0) to one full oscillation (rate = 1)—rather than testing only a few discrete jumps in the coefficients. Figure 5 illustrates different rates of change. We consider 21 rates ranging from zero to one full oscillation in the coefficients. The signal‐to‐noise ratio is constant over time, and all coefficients are standardized between zero and one. Refer to the ‘Simulation’ section in the Supplementary Material for more details.

#### 4.1.2 Sample size

We vary the study duration (14, 28 or 56 days) and the number of ESM beeps per day (6, 8 or 12), corresponding to study designs of 2, 4 or 8 weeks with 6, 8 or 12 measurements per day. We varied both conditions separately because the number of daily measurements directly affects the effective length of a 24‐h training window. This demonstrates how study design can implicitly affect training window size even when the window selection method remains the same. Although the number of measurements per day is higher than in regular ESM studies, phenotyping studies often have a higher assessment frequency (Jacobson & Feng, 2022). The resulting total time series length ranges from 84 to 672.

#### 4.1.3 Training window candidate library

We fit a library of candidate models ${𝒦}_{t}$ to the generated data that differ in their training window. Specifically, we fit 21 OLS models with training windows ranging from 5% to 25% of the sample size in increments of 1%. Additionally, we add standard selection models (last day, last two days, expanding window), bringing the library size to K=24 models. Based on all 24 models, we construct the average of all training window models and the dSL and eSL as described earlier. We implement this entire procedure for both OLS regression models and random forest models separately to test whether our results are robust to model choice. Due to the high computational demand of fitting RF models, we construct the average and dSL for both model types, but only construct the eSL for OLS models.

In sum, we vary the rate of change of the underlying model, the length of the time series through the number of days and measurements per day, the method to select the underlying (combination of) training window(s) and the underlying model type (OLS vs. RF).

We evaluate the predictive performance of the different selection methods by averaging the MSPE of the outer loop of the forward validation procedure. To construct the super learner, we require an estimate of the prediction errors of all candidate models using an inner validation loop. Our largest candidate model uses a training window of 25% of the available data, and we use an additional 5% of the data to obtain more stable error estimates for the candidate models. Therefore, we only use the remaining 70% of each time series to evaluate the final predictive performance by averaging the prediction error across this portion.

### 4.2 Results

#### 4.2.1 Ordinary least squares

**FIGURE 6:**
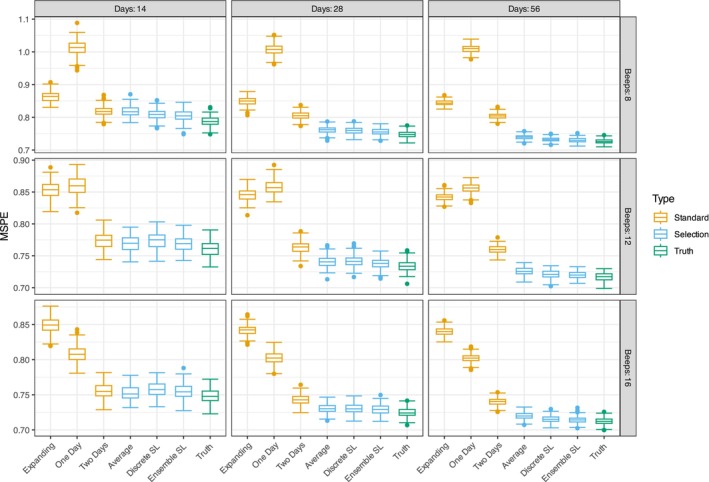
Depicts the MSPE of all model selection methods. Each column depicts a different condition for the study days (14, 28 and 56), while each row depicts the different number of beeps per day (8, 12 and 16). The standard methods are depicted in orange (i.e. one or last 2 days, expanding window), the selection methods that consider multiple windows in blue, and the best possible training window in green.

We evaluated the MSPE of the outer loops of all model selections by averaging them across all 200 simulation iterations and the simulation conditions. We start by describing the results for the OLS model. Figure 6 shows the prediction error for different window selection methods, numbers of days, and beeps per day. Each column represents a number of days, and each row a number of beeps per day. The *Standard* methods (orange) include the expanding window and windows of one and two days. The *Selection* methods (blue) consider multiple windows. The green boxplots, labelled ‘Truth’, represent the optimal predictive performance. This optimal performance could be achieved if the ideal training window minimizing MSPE were known. We determined it by calculating the error for each model on 100 independent test sets and selecting the best model at each time point.

Overall, prediction error decreases with more beeps and study days. However, the expanding method is an exception: it performs consistently and doesn't benefit from more data. The ensemble and discrete super learner and the simple average tend to achieve the lowest MSPE compared to standard methods. Their performances are nearly indistinguishable, except in the 56‐day condition, where the discrete and ensemble super learners perform better than the simple average. Increasing the number of days within each beep condition, we observe that the spread for standard methods (orange) decreases, yet their median MSPE remains relatively stable. In contrast, the selection methods (blue) benefit significantly from additional days, showing a clear decrease in prediction error. As expected, the super learner approaches the true optimal predictive ability (green), but it never exceeds it. This indicates some error in selecting the optimal training window.

**FIGURE 7:**
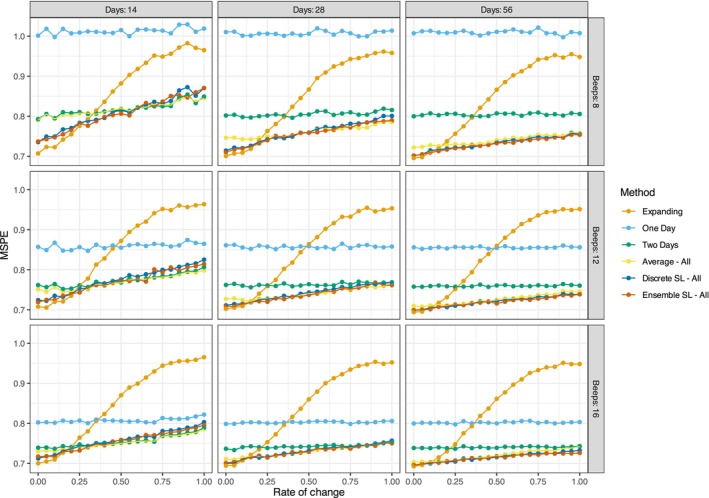
Depicts the MSPE value of one‐step‐ahead predictions and real values. Each column depicts a different condition for the study days (14, 28 and 56), while each row depicts the different number of beeps per day (8, 12 and 16).

Figure 7 shows prediction error broken down by the rate of change on the x‐axis. Unlike Figure 6, which displays MSPE values as boxplots, this figure represents the mean prediction errors of different methods using lines and points colour‐coded according to the legend on the right. The rate of change indicates how strongly the underlying coefficients varied over time; as shown in Figure 5, zero means no change, and one indicates a full oscillation over the time series. Across all conditions, the expanding window method (orange) performed best when there is no change (rate = 0) but experienced a sharp increase in error as the rate increases. This is expected because it assumes all historical data is equally useful, failing to account for coefficient changes over time. In general, all methods decrease in predictive performance as more change occurs, especially in the smallest sample size condition (14 days and 8 beeps).

As already evident from Figure 6, the super learners and the simple average of different training windows perform best across most conditions. These methods show relatively similar performance, but in conditions with low rates of change (0 to around 0.25) and fewer days (e.g. 14 days), the discrete and ensemble super learner slightly outperforms the simple average. This can be explained by the discrete learner selecting the expanding window under these conditions, which is the best‐performing method when changes are minimal. Additionally, the standard training window of 2 days (green) performs as well as the selection methods (average and discrete learner) only when we have 14 days of data. The performance gap between the 2‐day window and the selection methods is less pronounced in these scenarios. In conditions with more study days, the difference between the selection methods and the two‐day window becomes more apparent but somewhat diminishes in situations of higher change. In summary, the results from both plots indicate that adaptive methods like the discrete super learner and simple average tend to outperform standard methods like the expanding window or a two‐day training window, especially when the number of study days exceeds 14.

#### 4.2.2 Random forest

We now examine whether these findings generalize from OLS to non‐linear modeling approaches, namely RF models. Due to computational constraints, we did not calculate the true optimal predictive performance for RF models. Additionally, since the gap between the best window selection methods and the Truth benchmark was minimal in the OLS simulations, this omission is unlikely to affect our main conclusion for the RF models.

**FIGURE 8:**
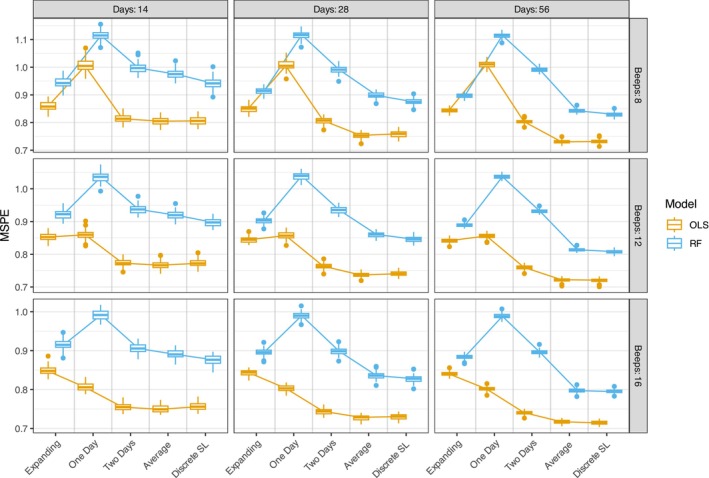
Depiction of the MSPE of all model selection methods and comparison between the OLS (orange) versus the RF (blue) models. Each column depicts a different condition for the study days (14, 28 and 56), while each row depicts the different number of beeps per day (8, 12 and 16). MSPE, mean squared prediction error; OLS, ordinary least squares; RF, random forest.

Figure 8 shows the prediction error for different window selection methods, numbers of days, and beeps per day and compares the results of the OLS models (orange) and the RF models (blue). The Random Forest models consistently exhibit higher prediction errors than OLS models across all conditions, which is expected given that the data‐generating process follows a linear relationship that favors OLS. However, the relative performance patterns of window selection methods remain largely consistent between model types. The averaging and discrete super learner methods continue to outperform single‐window approaches in RF models, with the performance advantages being even more pronounced than in OLS models. Notably, the RF models appear to perform worse with limited training data, as the training windows of 1 and 2 days consistently show the poorest results across all conditions, likely due to these models underfitting the data. Unlike OLS models, the expanding window method in RF shows a larger improvement with longer study durations, and is almost on par with the average and the discrete SL in the 8‐beep condition. Additionally, the discrete SL seems to have a larger advantage over the average for the RF models, while they were very similar in performance for the OLS models. These findings suggest that while the absolute performance differs between model types, the benefit of considering multiple window sizes using the average or the discrete super learner over fixed single‐window approaches holds across both linear and non‐linear models. These results reinforce our recommendation to use multiple training windows and average their predictions.

Figures 3–5 in the Supplementary Material further break down the prediction error by the rate at which the coefficients change over time. These figures confirm once again that averaging and discrete super learner methods outperform single training window approaches. The one‐day window in particular shows poor predictive performance, regardless of how quickly the coefficients change over time for the RF models, indicating underfitting. For RF, the expanding window performs well when change is slow but is overtaken by the averaging method at faster rates of coefficient change—although this crossover occurs more gradually than in the OLS models. The discrete super learner maintains a larger advantage over averaging in RF models, though the performance gap becomes smaller as study duration and beep frequency increase.

In summary, the simulation results show that considering multiple training windows simultaneously, either through averaging or a super‐learning approach, improves predictive performance across varying rates of non‐stationarity in the data‐generating process. To test whether this finding translates to practice, the following empirical example examines a dataset that is typical of digital phenotyping research, in which a single training window is usually employed. While the simulations used both OLS and RF, in the empirical example, we use only a random forest because the number of covariates is too large for OLS.

## 5 EMPIRICAL EXAMPLE

In this section, we demonstrate the application of the super learning procedure using data from two studies involving 21 university students, each lasting 28 days. The data includes both ESM data on affect and passive sensor data from participants' smartphones. For more detailed information on the study design, please refer to Langener, Bringmann, et al. (2024) and Langener, Stulp, et al. (2024).

### 5.1 Data

The ESM data were collected through the m‐Path application (Mestdagh et al., 2023), while the passive sensing data were gathered via the Behapp app (Jagesar et al., 2021). In this analysis, we predicted both negative and positive affect as outcome variables. Positive affect was computed as the mean of the responses to the ESM items ‘I feel happy’, ‘I feel energetic’ and ‘I feel relaxed’. Negative affect was computed as the mean of the items ‘I feel sad’, ‘I feel anxious’, ‘I feel stressed’ and ‘I feel irritated’. All items were rated on an 11‐point Likert scale, ranging from ‘I strongly disagree’ to ‘I strongly agree’.

As predictors, we used features calculated from the passive sensor data over the past 3, 6, 12 and 24 h.^1^ As they are measured at a much higher frequency than the ESM questionnaires, they need to be aggregated into features. These features included time spent on specific apps and time spent at specific locations as recorded by GPS.^2^ Regarding pre‐processing, we excluded covariates with a variance close to zero and also imputed missing values via the median.^3^

### 5.2 Model specification

We used a random forest prediction model (Breiman, 2001) because, in digital phenotyping research, it is more frequently used than linear methods (Benoit et al., 2020; Opoku Asare et al., 2021). This is because it can handle a large number of covariates (Biau, 2012), non‐linearity as well as interactions (Couronné et al., 2018). Additionally, it can also achieve high predictive performance without the need for fine‐tuning (Probst et al., 2019). Thus, our analysis deviates from our simulation study as we used a small number of covariates and linear change for illustrative purposes. Additionally, the OLS model was omitted as it cannot handle the large number digital phenotyping features. To address potential non‐stationarity, we adopted a procedure very similar to that used in the simulation. Specifically, we fitted several models that varied in the size of their training window. The size of these training windows was based on the average number of ESM assessments collected per individual, which was 154. The smallest window included 7 assessments (5 percent of 154), while the largest window used 36 assessments (25 percent of 154^4^). The intermediate windows increased incrementally by one percent each, resulting in a total of 21 windows. In addition to these models, we incorporated standard selection methods with fixed window sizes based on the last one or two days of observations, which corresponded to windows of 5 and 10 assessments, respectively, as participants were prompted at least five times per day. We also tested an expanding window method that incorporated all available past data. Thus, the final candidate library ${𝒦}_{t}$ included 24 models that are considered when constructing the more advanced selection methods.

### 5.3 Results

**FIGURE 9:**
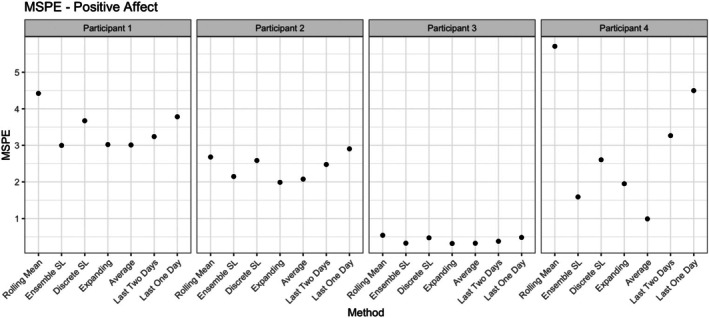
Mean squared prediction error (MSPE) for positive affect for four example participants across selection methods. The relatively lower prediction error of Participant 3 might be the artifact of the overall lower variance in their time series compared to the other participants. See Figure S8 in the Supplement. MSPE, mean squared prediction error.

For the average selection method, we computed the mean of the predictions from all models in the library ${𝒦}_{t}$. To assess the performance of the super learners, we employed forward validation as previously described and computed the MSPE across all outer validation folds. The average mean squared prediction error (MSPE) across all outer validation folds for four example participants is displayed in Figure 9 for positive affect. Additionally, Table 1 presents the average MSPE across all participants, along with the average rank of each method, determined by ordering the MSPE values in ascending order and then averaging the ranks across participants.

**TABLE 1:** Mean squared prediction error (MSPE) and average rank of all model selection methods from the digital phenotyping study sample. Both metrics are averaged across all participants. For each column, the model selection method(s) with the best predictive performance is (are) indicated in bold. For both MSPE and average rank, a lower value indicates a better performance.

Method	Negative affect		Positive affect	
	MSPE	Avg. rank	MSPE	Avg. rank
Last one day	1.91	4.19	2.78	5.24
Last two days	1.81	3.81	2.48	3.90
Expanding	1.83	4.24	2.40	3.14
Average	**1.72**	**2.76**	**2.37**	**3.10**
Discrete SL	1.84	4.43	2.70	4.57
Ensemble SL	1.75	3.19	**2.37**	3.29
Rolling mean	2.15	5.38	2.79	4.76

Figure 9 gives insight into the variance of the selection methods between participants. While the eSL and the average, as well as the expanding window, tend to perform rather well, this is not the case for all participants. For example, the expanding window is the worst performing one for Participant 4. Additionally, the differences between all methods vary and are, for example, very small for Participants 2 and 3. To determine how the average prediction methods compare to each other, we now turn to the summary table.

Table 1 presents the results of the forward validation. The simple average of all candidate models performed best, achieving the lowest MSPE for both negative affect (1.72) and positive affect (2.37), and the highest average rank across all methods. The ensemble super learner performed similarly well, especially for positive affect, where it matched the average method's MSPE (2.37).

Among the standard selection methods, the last two days and the expanding window methods performed relatively well but had higher prediction errors compared to the average and ensemble approaches. The discrete super learner, however, did not perform as well, ranking between fourth and fifth in terms of both MSPE and average rank, only outperforming the last 1‐day method. The baseline model generally performed worse than the other models in terms of both rank and average error. However, especially in comparison to the last day method for positive affect, the MSPE of the rolling mean is almost the same, and the average has a higher rank. This highlights the importance of choosing appropriate training windows to meaningfully outperform simple baseline methods, but also highlights that a simple baseline might be better in some cases.

These results suggest that the average method is a simple way to select a well‐performing window, as it is only rarely outperformed by other methods in the empirical example. They also indicate that combining predictions from multiple training windows (as done in the average and ensemble methods) often outperforms strategies that rely on selecting a single ‘best’ window. This holds regardless of how the single window is chosen, whether based on theoretical reasoning (such as using all available data in the expanding window approach) or empirical performance (such as choosing the window with the lowest prediction error in the discrete learner approach).

## 6 DISCUSSION

In this study, we aimed to find a solution to the problem of how to select the optimal training window (combination) that improves the predictive accuracy in a real‐time setting under non‐stationarity. For this purpose, we compared the predictive performance of several common and new training window selection methods, fitting both linear (OLS) and non‐linear models (RF). The simulation study shows that using multiple training windows generally improves accuracy compared to single‐window methods, such as the frequently used 24‐h window. However, the differences between the simple averaging method and more complex super learner approaches were largely negligible.

In the empirical example, the average and ensemble super learner also performed better than the single window methods, but the advantage was smaller. Surprisingly, the discrete super learner performed worse than all but the 24‐h window method, which contrasts with prior recommendations that suggested using this approach to select the optimal training window (Verachtert et al., 2022). Overall, the gains in predictive accuracy of the super learner are minimal compared to the simple average. Additionally, constructing the super learner requires the complex forward validation approach, which is unavailable in standard software (e.g., *caret* package, Kuhn, 2008). Thus, we suggest that applied researchers consider multiple training windows and average their predictions rather than relying on a single best window. This recommendation aligns with findings from the M4 forecasting competition (Makridakis et al., 2020).

Like any simulation study, many parameters had to remain fixed, and therefore, the conclusions of our study are subject to a number of limitations. We focused exclusively on conditions where the relationship between sensor data and outcomes changed gradually. In real‐world scenarios, however, individuals may experience abrupt shifts in these relationships – such as when they go on vacation, begin a new intervention or relocate to a different city. Future research should address these limitations by simulating abrupt changes and testing the impact of training windows in empirical samples with (known) moments of abrupt change.

Another limitation is that our model libraries lacked algorithmic diversity. In the simulation study, we used the OLS and RF models, whereas in the empirical application, we only used the RF model. Although we tested the predictive accuracy of both algorithm types, we did not include them in a single model library or combined their predictions. This approach contrasts with recommendations on the use of super learner procedures, which suggest using ‘a rich library of algorithms that is diverse in its learning strategies’ (Phillips et al., 2023) to improve predictive accuracy. A related limitation is that our simulations only considered linear relationships between predictors and outcomes, which may not reflect the complexity of real‐world relationships between sensor data and psychological outcomes. Future research should address both issues by (1) combining diverse prediction algorithms with varying training window sizes and (2) simulating non‐linear data‐generating processes.

Our study highlights the potential of adaptive training window selection to improve predictive accuracy but faces technical obstacles that currently limit implementation in a real‐time setting (Van Genugten et al., 2025). Current advances in mobile computing, such as the *TinyML* approach, make the deployment of machine learning models on resource‐constrained devices like smartphones and smartwatches possible (Heydari & Mahmoud, 2025), including stress detection using sensor data (Abu‐Samah et al., 2025). However, these approaches mainly use pretrained, non‐personalized algorithms that do not require continuous updating, unlike our suggested approach. While personalization of models could be achieved by offloading the processing to a server instead (Osia et al., 2020), this strategy introduces additional computational and infrastructural overhead. As an alternative to the detection approach we investigated, one could reduce the required computation speed by predicting outcomes further into the future. For instance, Stamatis et al. (2024) repeatedly predicted depression symptoms at the same time as well as 1 and 2 weeks in advance. This longer prediction horizon would allow sufficient time buffers for both model retraining and potential intervention delivery in near real time when processing is offloaded to the server.

In sum, this study was motivated by the potential applications of real‐time monitoring and prediction of mental health symptoms using ESM and passive sensing data. As selecting the appropriate window size is key given the issue of non‐stationarity, we compared several methods of selecting the optimal training window approach and made practical recommendations: We encourage researchers seeking accurate predictions to consider multiple training windows and, ideally, to average the predictions across those windows rather than relying on a single model.

## AUTHOR CONTRIBUTIONS

**Fridtjof Petersen:** conceptualization; methodology; software; writing – original draft; writing – review and editing; project administration; visualization. **Jonas M. B. Haslbeck:** writing – review and editing; conceptualization; methodology; supervision. **Jorge N. Tendeiro:** writing – review and editing; supervision; methodology; conceptualization. **Anna M. Langener:** writing – review and editing; data curation; formal analysis; methodology; visualization. **Martien J. H. Kas:** data curation; writing – review and editing. **Dimitris Rizopoulos:** conceptualization; methodology; software; supervision; project administration; writing – review and editing; funding acquisition. **Laura F. Bringmann:** conceptualization; methodology; supervision; project administration; writing – review and editing; funding acquisition.

## CONFLICTS OF INTEREST

The authors declaure that they have no known competing financial interests or personal relationships that could have appeared to influence the work reported in this paper.

## DISCLOSURE OF ARTIFICIAL INTELLIGENCE‐GENERATED CONTENT (AIGC) TOOLS

The authors did not use any (AIGC) tools.

## Supporting information




Data S1

## Data Availability

The data that support the findings of this simulation study are openly available in the repository ‘Simulation Code for “Comparing Training Window Selection Methods for Prediction in Non‐Stationarty Time Series”’ at https://osf.io/bmne5/?view_only=439474e2dcf9455ca0583ebcd5dcf6d9.

## Footnotes

^1^
Note that depending on the aggregation window length, the distribution of the features can change considerably. In Figures S9 and S10 in the supplement, we have included an illustrative example of how the skew of the distribution can change depending on the aggregation window for two participants. For more information on how the aggregation window of raw data can impact the relationship with the outcome and model performance, see the relevant literature (Hu et al., 2015; Langener, Stulp, et al., 2024; Liu et al., 2022; Quisel et al., 2017; Stamatis et al., 2024).

^2^
A complete list of all covariates is included in the Supplement as Tables S1 and S2.

^3^
Note that more sophisticated imputation strategies such as multiple imputation could be used alternatively (Ji et al., 2018), but given the relatively low missingness rates and our focus on training window selection rather than imputation methodology, median imputation was deemed sufficient for our purposes. More general discussions of handling missingness in digital phenotyping data exist in the literature (Kiang et al., 2021; Langener, Stulp, et al., 2024; Leaning et al., 2024, 2025; Shen et al., 2024).

^4^
We capped the training window at 25 percent of the data, as an additional 5 percent was needed to construct the super learners, which rely on an initial estimate of prediction error. This left us with 70 percent of the data that we could use to compute the prediction error for all selection methods. Increasing the size of the training windows even more could decrease the accuracy of the estimated prediction error.
