# Supplementary material for: Comparing training window selection methods for prediction in non‐stationary time series
Source: Br J Math Stat Psychol. 2026 Jan 13;79(2):341–61. doi: 10.1111/bmsp.70018 (PMC13067991; doi:10.1111/bmsp.70018)
Supplement: Supplementary file 1 — Data S1 [file BMSP-79-341-s001.pdf]

Supplementary Material

Window selection methods

Standard methods

One common method for selecting a training window is the *expanding window* method, which we denote as $f_{\text{exp}}(\cdot)$. In this approach, all previous data up to the current time point are used to train the model, eliminating the need to decide on the specific size of the window as data is never discarded (e.g., Balliu et al., 2024). Using our framework, this method is defined by setting the training window size equal to the current time point, $w_k = t$, when training the model. This is illustrated in Figure S1 on the left side in row 3, where the model predicts time point 7. As the corresponding training window is $w_k = t = 7$, the model is estimated using all the previous data: $D_k = \{y_1, \ldots, y_6;\ \boldsymbol{x}_1, \ldots, \boldsymbol{x}_6\}$.

Figure S1

Illustration of one-step-ahead predictions (red) for models trained on all past data (yellow) and the last four data points (blue).

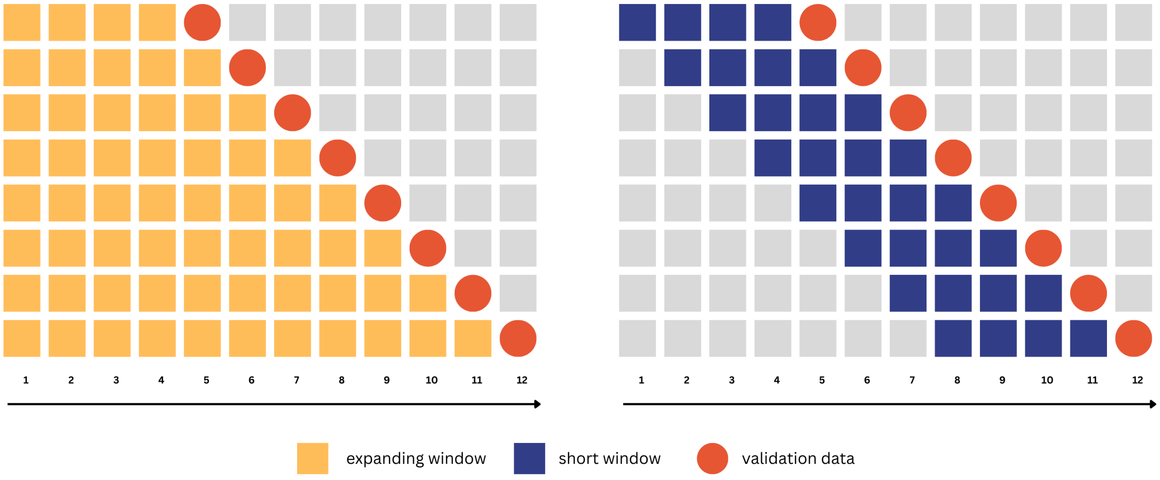

Another approach is the *last day window* method, which selects the training window based on the number of ESM signals obtained per day. This heuristic-based method, which we denote as $f_{24}(\cdot)$, aims to account for potential changes in the underlying relationship over time by discarding data older than a day. In Figure S1 on the right side, four ESM assessments were scheduled per day; therefore, the training window consists of $w_k = 4$.

Both window selection methods are heuristic-based. The expanding window assumes a constant data-generating mechanism, while the last-day window uses a fixed 24-hour period for all individuals, presuming it effectively captures the underlying change. These approaches are likely suboptimal, because they do not account for heterogeneity across individuals and make assumptions on the rate of change, which may fail to reflect the datas real dynamic nature. Additionally, researchers often apply these methods without comparing them to alternatives. To address these issues, we introduce methods that utilize the entire library of training windows.

Average

Relying on a single model based on heuristics or unchecked assumptions - such as assuming stationarity in the expanding window method - can negatively impact predictive performance if the chosen model is misspecified and better-performing models are available. Averaging predictions across all models mitigates the risk of selecting an inadequate model by considering multiple plausible alternatives. One straightforward approach is to take an average of the predictions from all models in the library. This method accounts for the uncertainty in choosing the single best model and can reduce the risk of selecting a poorly performing model. It is motivated by findings from studies like the M4 forecasting competition, where simple averages often outperform individual models or more sophisticated combinations of multiple models (Makridakis, Spiliotis, & Assimakopoulos, 2020). Formally, it is computed as a non-weighted average of all candidate models in the library $\mathcal{K}_t$, which may include the expanding or 24-hour windows discussed previously:

$$\hat{y}_{t+1} = \hat{f}_{\text{avg}}(x_{t+1}) = \frac{1}{K}\sum_{k=1}^{K}\hat{f}_k(\boldsymbol{x}_{t+1}, \hat{\boldsymbol{\beta}}_{kt} \mid D_k).$$

However, simple averaging treats all models the same, regardless of their individual predictive performance, as well-performing models are weighted equally with poorly performing ones. Super learning improves upon simple averaging by assigning greater weights to models with higher predictive accuracy. In what follows, we first define how we can quantify the predictive performance of all candidate models and subsequently introduce the super learner approach that is based on this metric.

Super learner

Super learning is a general method to combine different prediction models or algorithms to optimize the accuracy of predictions for an outcome of interest. The optimal combination of models is usually achieved by minimizing the prediction error, such as the mean squared prediction error (MSPE), of all candidate models, which measures how well models predict (i.e., the predictive performance). In general, the MSPE for a given model is defined as:

$$\text{MSPE} = E\left[\left\{Y_{t+1} - \hat{f}(\boldsymbol{X}_{t+1}, \hat{\boldsymbol{\beta}})\right\}^2\right], \tag{1}$$

where the expectation is taken with respect to the true data-generating distribution. Note that in (1), we have used a generic definition of the predictions $\hat{Y}_{t+1} = \hat{f}(\boldsymbol{X}_{t+1}, \hat{\boldsymbol{\beta}})$ as it describes the predictive ability of any procedures that obtain predictions. This includes evaluating models that make up the library $\mathcal{K}_t$ which consist of individual training window models, as well as procedures such as the average or the super learner, which are based on multiple individual models.

In practice, however, we do not have access to the true MSPE value of a model, as the data-generating distribution $f_t(\boldsymbol{X}_t, \boldsymbol{\beta}_t^*)$, which produces our observations that we wish to compare our predictions against, is unknown. Instead, we have to create an objective estimate of the error, denoted as $\widehat{\text{MSPE}}$, for which cross-validation is often used to account for overfitting. The specific cross-validation procedure used in this paper is discussed in the section "Forward Validation". The estimated $\widehat{\text{MSPE}}$ is then used to construct the super learner by combining the models in the candidate library $\mathcal{K}_t$, which correspond to OLS models with different training windows w_k.

First, we describe the *discrete super learner* (dSL), which selects the single best model based on cross-validated performance. Subsequently, we introduce the *ensemble super learner* (eSL), which combines the predictions of all candidate models by creating a weighted average. The ensemble super learner was introduced by Van Der Laan, Polley, and Hubbard (2007) and theoretically performs at least as well as the best single model among all candidate models.

To construct the dSL, we choose the model that minimizes the cross-validated $\widehat{\text{MSPE}}$, where k^* indicates the index of the selected model and $\widehat{\text{MSPE}}_k$ the cross-validated estimate of the mean square prediction error of the k-th model from the model library $\mathcal{K}_t$:

$$k^* = \underset{k=1,\ldots,K}{\text{argmin}}\, \widehat{\text{MSPE}}_k.$$

The prediction of this model theoretically minimizes the expected prediction error $\widehat{\text{MSPE}}_k$ for future data:

$$\hat{y}_{t+1} = \hat{f}_{\text{dSL}}(x_{t+1}, \hat{\theta}_{k^*}), \tag{2}$$

where $\hat{\theta}_{k^*}$ denotes the parameters of the chosen model.

In contrast to the dSL, the eSL combines the predictions of all candidate models by creating a weighted average, where weights are determined based on each models cross-validated performance in such a way that they minimize the overall predictive error. The model weights ω have a simplex constraint as they take values between 0 and 1 and sum to 1. They are optimized in the following way:

$$\hat{\omega}_k = \min_{\omega_k \in \mathcal{S}_1^K} \sum_{k=1}^{K} \omega_k \widehat{\text{MSPE}}_k.$$

The corresponding ensemble prediction for the next data point takes the form:

$$\hat{y}_{t+1} = \hat{f}_{\text{eSL}}(x_{t+1}) = \sum_{k=1}^{K} \hat{\omega}_k \hat{f}(x_{t+1}, \hat{\theta} \mid D_k), \tag{3}$$

where $\hat{\theta}_k$ denotes the parameters of the k-th model trained on data D_k. By assigning higher weights to models with better predictive performance, the eSL seeks to enhance prediction accuracy beyond what is achievable by individual models or simple averaging.

Forward validation

However, to evaluate the predictive performance of the super learner itself - not just the candidate models - we introduce the *forward validation* approach. It allows us to test whether the super learners improve predictive performance. This nested time series cross-validation method consists of an outer and inner loop, where both loops perform a time series cross-validation, iteratively splitting the data into sequential training and validation sets (see Figure S2 for an illustration). In the outer loop (left side), we evaluate the super learners predictive accuracy by testing it on validation data (red) that was not part of its construction. Each training set in the outer loop (orange) undergoes a further inner loop of cross-validation; the error of the candidate models is estimated on a reduced dataset. As each outer loop fold grows in size and we repeat the inner loop each time, it allows us to reconstruct the super learner for every new data point that we collect and adapt to changes in the predictive ability of the candidate learners.

Figure S2

Example of forward validation. The inner loop estimates prediction errors for different candidate models and constructs super learners. The outer loop is used to estimate the prediction error of super learners as well as candidate models. On the right panel, you can see an example of how the first outer fold is split into an internal validation loop.

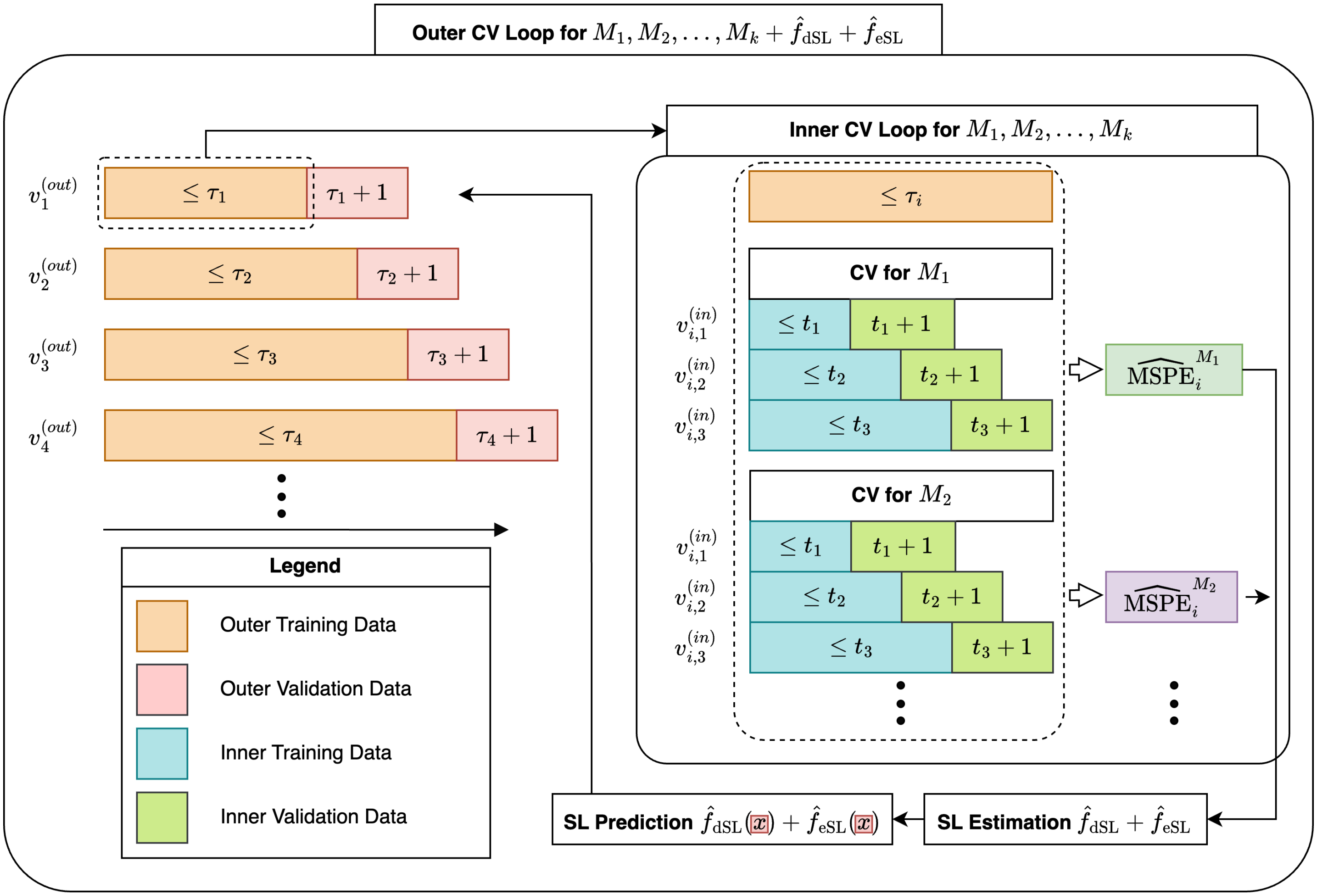

The outer loop is depicted on the left panel of Figure S2, with each fold represented by a single row. The outer training data (orange segments) estimate the candidate models' errors and constructs the super learners. The red segment represents the outer validation data, used to test the performance of all models, including the discrete and ensemble super learners. The entire collection of outer folds is denoted by $V^{(\text{out})} = \{v_1^{(\text{out})}, \ldots, v_I^{(\text{out})}\}$ and has size I. The number of outer folds I is determined by the total length of the time series T and the minimum number of observations L needed to perform the inner loop, calculated as $I = T - L$. The minimum number of time points L is a combination of the largest training window, as well as the amount of error estimates needed from the inner loop to estimate the first super learner. Within each fold, the outer training data (orange) includes all observations up to and including the specific time point τ_i, represented as $t \leq \tau_i$, since the time series length changes depending on the specific loop. Each folds outer validation set (red)

consists of the observation at the next time point $\tau_i + 1$. For each outer training set, an inner loop is constructed.

The inner loop, illustrated on the right panel of Figure S2, estimates the prediction error for each candidate model $\mathcal{M}_k$. Note that the inner loop is performed repeatedly and separately for each outer fold. For a given outer fold $v_i^{(\text{out})}$, the corresponding collection of inner folds is denoted as $V_i^{(\text{in})} = \{v_{i,1}^{(\text{in})}, \ldots, v_{i,J_i}^{(\text{in})}\}$, where $J_i = \tau_i - M$ denotes the number of inner folds for this specific outer loop. M denotes the minimum number of observations needed to perform the first inner fold and corresponds to the largest training window of the candidate models, as this is the minimum number of data points needed to train the first model. For each inner fold $v_{i,j}^{(\text{in})}$, the inner training set (blue) includes all data up to and including time point t_j, where $t_j = M + j$ for $j = 1, \ldots, J_i$. The inner validation set (green) consists of the observation at the next time point $t_j + 1$.

To estimate the prediction error for each model $\mathcal{M}_k$ in the inner loop, the model is trained on the inner training set $D_k^{i,j} = \{y_{t-w_k+1:t}, \boldsymbol{x}_{t-w_k+1:t}\}$ with $t = t_j = M + j$, which results in the corresponding prediction function $f_k^{i,j}$. The prediction function then produces a one-step-ahead prediction for the inner validation time point y_{t_j+1}. The prediction error for each inner fold is computed as the squared difference between the predicted value and the actual observed value at the validation time point. By averaging these errors across all folds, we obtain an estimate for the MSPE for the specific i-th outer fold and k-th model:

$$\widehat{\text{MSPE}}_i^{(\text{in},k)} = \frac{1}{J_i} \sum_{j=1}^{J_i} \left\{ y_{t_j+1} - f_k^{i,j}(\boldsymbol{x}_{t_j+1}, \hat{\theta} \mid D_k^{i,j}) \right\}^2 . \tag{4}$$

As a result of performing a single inner loop, we obtain an estimated prediction error of all candidate models, and we can now construct both the discrete and ensemble super learner as described in (2) and (3) as well as retrain all candidate models on the entire outer training data of the outer loop. This leaves us with a complete model library denoted by $C_t = \{\mathcal{M}_1, \ldots, \mathcal{M}_k, \hat{f}_{\text{avg}}, \hat{f}_{\text{dSL}}, \hat{f}_{\text{eSL}}\}$ of size $K + 3$. Note that we explicitly included the average selection method in the library, as this also combines different training windows of the candidate library $\mathcal{K}_t$. The last day and expanding window selection methods, however, are part of the candidate library as they represent a constant and static choice of training windows.

Using the entire model library C_t, we can predict the outer validation point at $\tau_i + 1$ and calculate the prediction error for all models, including the super learner. By averaging the prediction errors across all outer folds, we obtain an estimate of the out-of-sample prediction error $\widehat{\text{MSPE}}_{(\text{out})}^{C}$ for each model in the complete library C_t, allowing us to compare whether the super learners perform better than any given candidate model:

$$\widehat{\text{MSPE}}_{(out)}^{\mathcal{M}_c} = \sum_{i=1}^{V_{out}} \{y_{\tau_i+1} - \hat{f}_c(x_{\tau_i+1})\}^2 , \tag{5}$$

where $\hat{f}_c(\cdot)$ denotes which model of the full model library C_t was used for prediction.

Simulation

This section explains how the time-varying coefficients $\boldsymbol{\beta}_t$ were generated in more detail.

The coefficients $\boldsymbol{\beta}_t$ change over time following a sine function, with the rate of change determining how quickly they vary. Each covariate $X_{i,t}$ is independently drawn from $\mathcal{N}(0, 1)$. The

time points t_i span from 0 to $2\pi \times$ rate where the rate expresses how quickly the coefficients change and determines the number of complete sine wave cycles over the T time points. The time points are calculated using the formula:

$$t_i = \frac{2\pi \times \text{rate} \times (i-1)}{T-1}, \quad \text{for } i = 1, 2, \ldots, T\,. \tag{6}$$

Secondly, we generate the coefficients for each time point t_i and each covariate j:

$$\beta_{i,j} = \sin\left(t_i + \phi_j\right)\,,$$

where the phase shift ϕ_j for each covariate is:

$$\phi_j = \frac{2\pi(j-1)}{p}, \quad \text{for } j = 1, 2, \ldots, p\,.$$

The phase shift is introduced so that not all covariates have the same coefficient value over time; consequently, the sum of squares of coefficients stays constant over time. Additionally, we normalized all coefficients to stay in the range of [0, 1] by applying min-max scaling across all coefficients. The normalization formula used was $\beta_{i,j} = \frac{\beta_{i,j} - \beta_{\min}}{\beta_{\max} - \beta_{\min}}$, where $\beta_{\min}$ and $\beta_{\max}$ are the minimum and maximum values of $\beta_{i,j}$ across all time points and covariates.

To focus solely on the rate of change of the underlying data-generating mechanismand not on the predictive ability over time as a function of the changing coefficientsit is important to fix the signal-to-noise ratio (SNR) to be constant over time. Since all covariates are generated independently from a standard normal distribution, the variance of the signal depends on the sum of squares of the coefficients:

$$\text{Var}\left(\textstyle\sum_{j=1}^{P} \beta_{i,j} X_{i,j}\right) = \textstyle\sum_{j=1}^{P} (\beta_{i,j})^2\,, \tag{7}$$

which we kept constant. As we have four covariates and set the variance of the error term to $\sigma^2 = 0.75$ to achieve a constant signal-to-noise ratio over time as:

$$\text{SNR} = \frac{\text{Var(Signal)}}{\text{Var(Noise)}} = \frac{\sum_{j=1}^{P} (\beta_{i,j})^2}{\sigma^2} = \frac{1.5}{0.75} = 2\,.$$

Results: Random forest

Figure S3

Depiction of the MSPE of all model selection methods and comparison the OLS vs the RF models. The first column depicts the result for the OLS model, while the second column shown the RF results. Each row depicts the different number of beeps per day (8,12,16), while the x-axis depicts the different rates of change. The results in this plot are shown for the 14 Days condition.

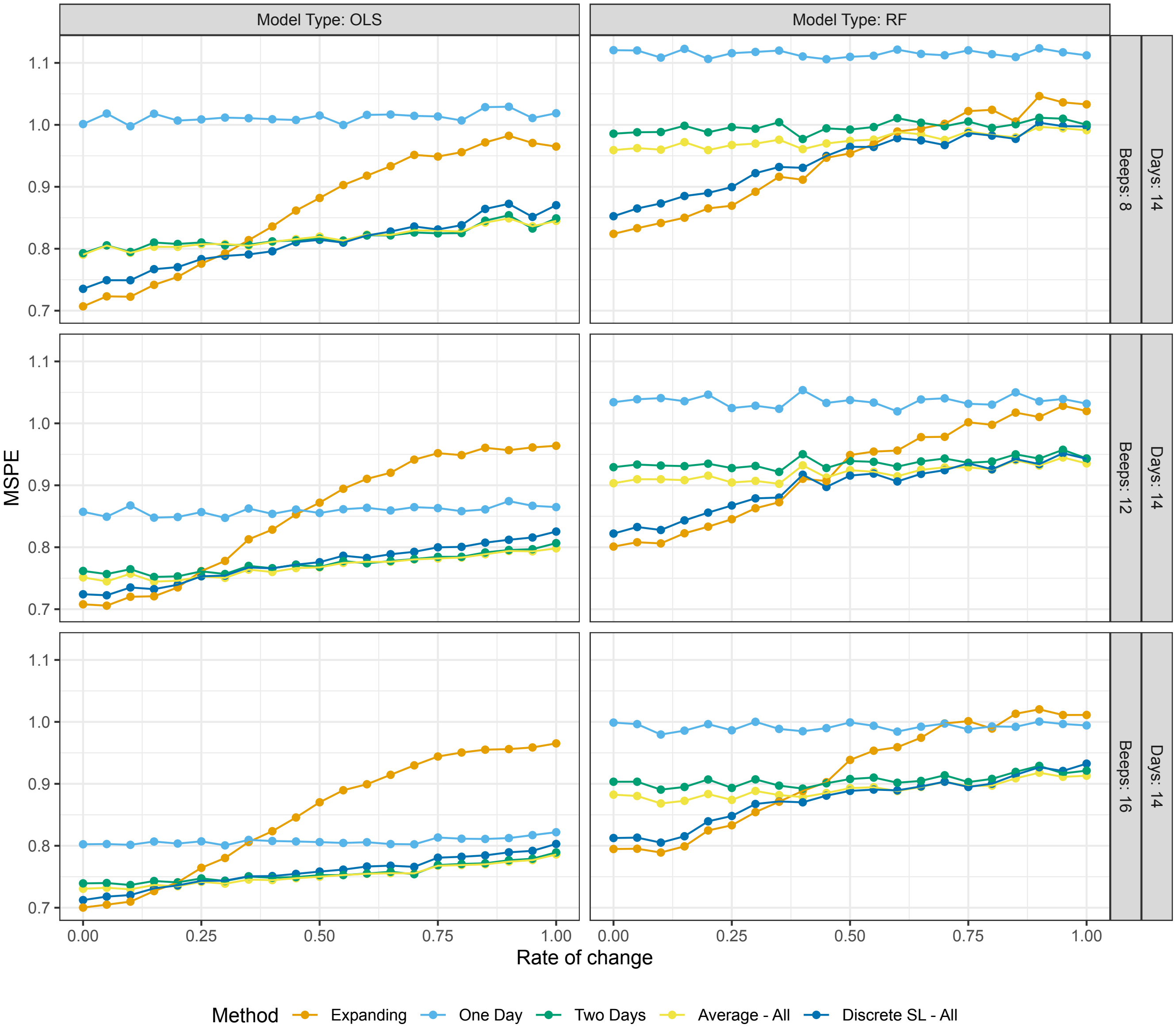

Figure S4

Depiction of the MSPE of all model selection methods and comparison the OLS vs the RF models. The first column depicts the result for the OLS model, while the second column shown the RF results. Each row depicts the different number of beeps per day (8,12,16), while the x-axis depicts the different rates of change. The results in this plot are shown for the 28 Days condition.

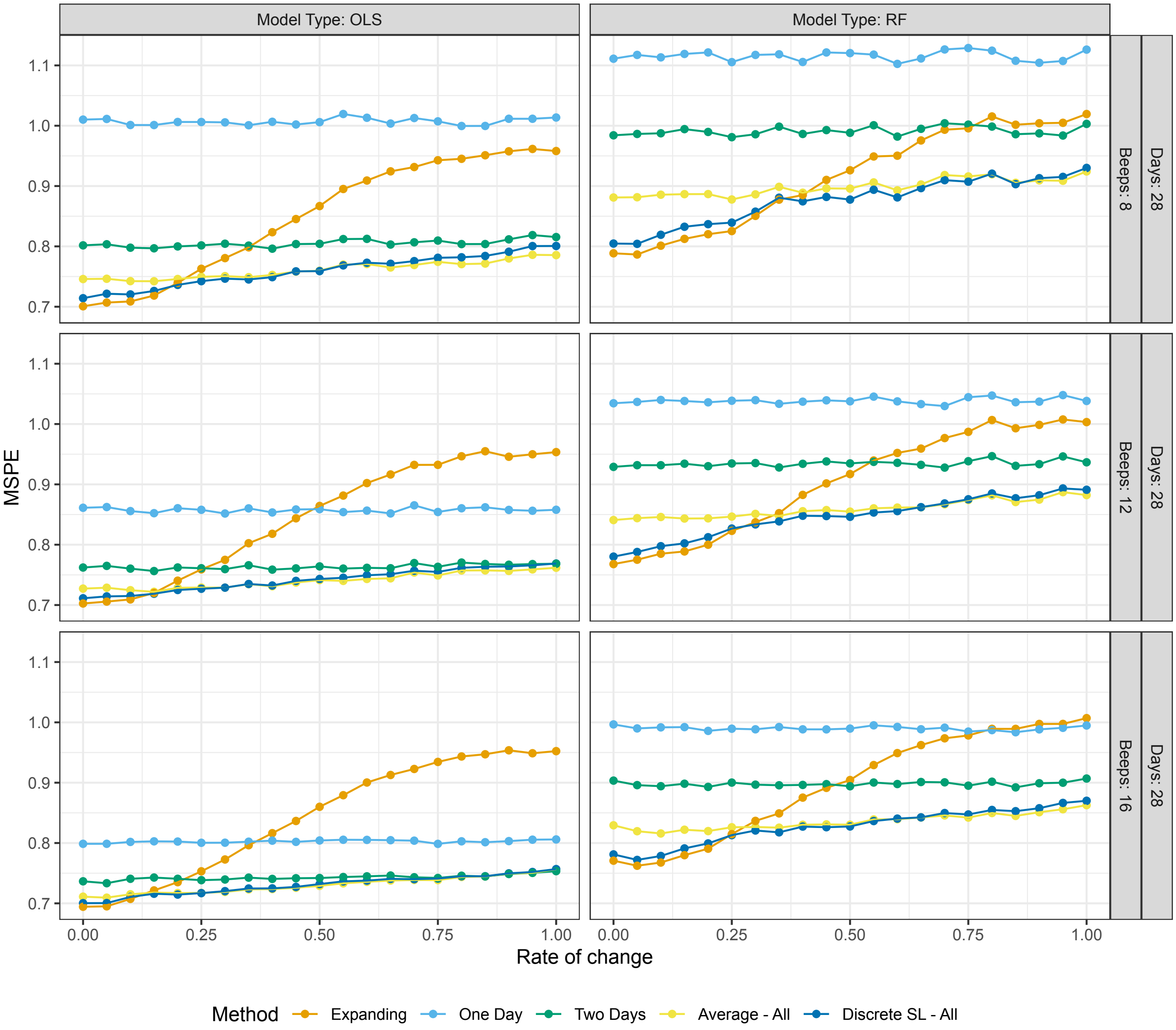

Figure S5

Depiction of the MSPE of all model selection methods and comparison the OLS vs the RF models. The first column depicts the result for the OLS model, while the second column shown the RF results. Each row depicts the different number of beeps per day (8,12,16), while the x-axis depicts the different rates of change. The results in this plot are shown for the 56 Days condition.

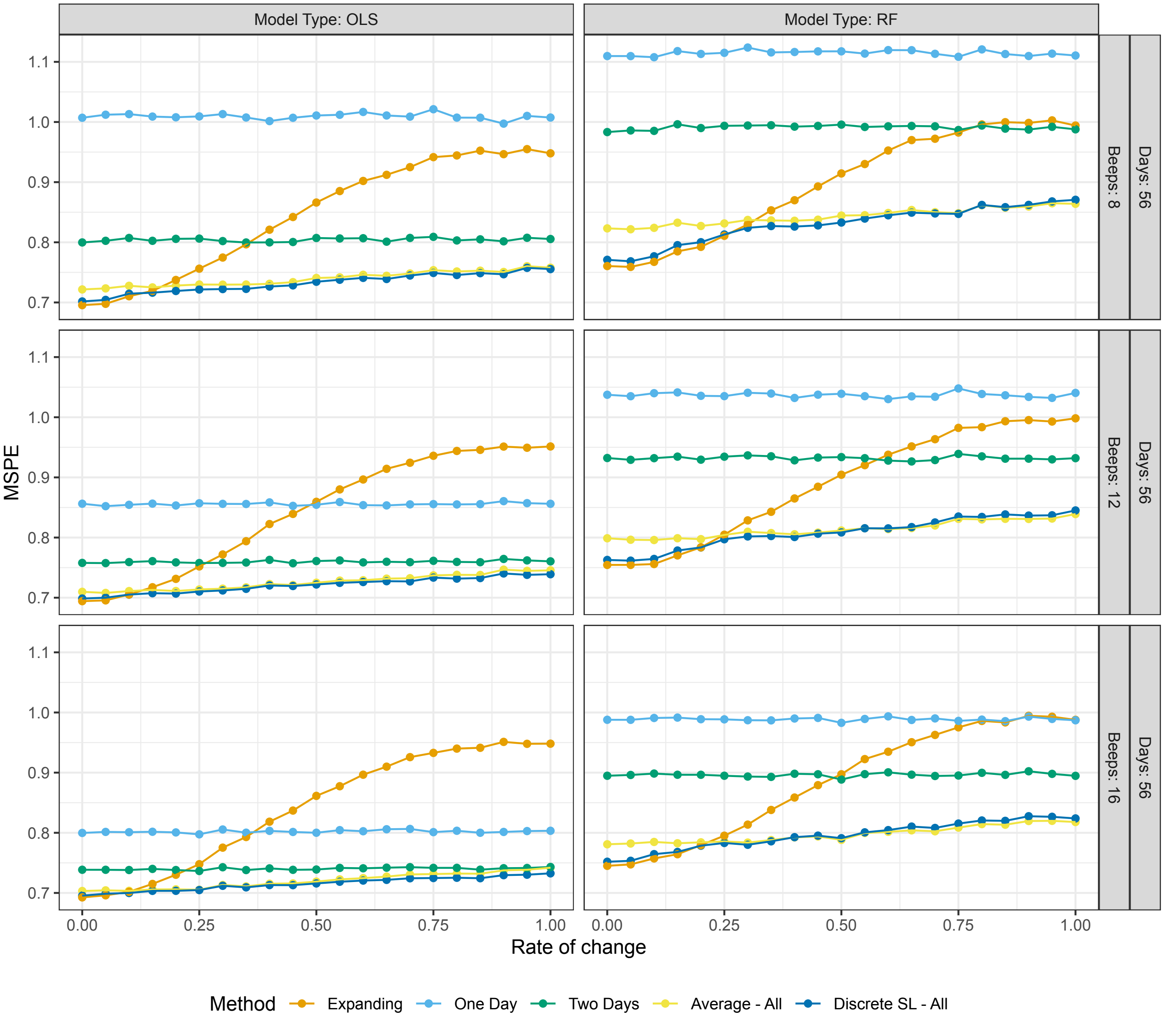

Empirical example

In Figure S7 and Figure S6, we can see the mean squared prediction error per participant for negative and positive affect, respectively.

Figure S6

Mean squared prediction error (MSPE) for negative affect for every participant across selection methods.

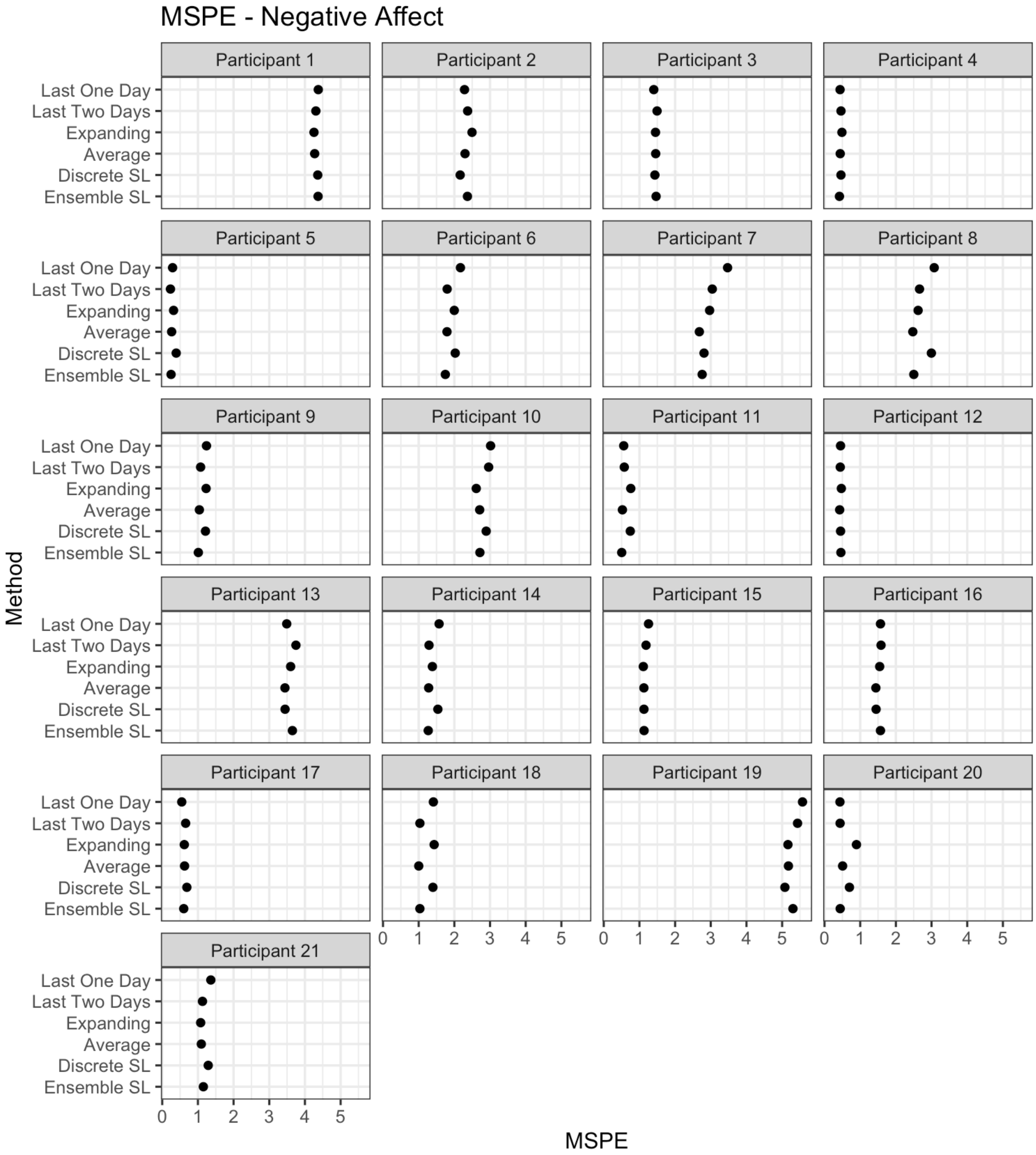

Figure S7

Mean squared prediction error (MSPE) for positive affect for every participant across selection methods.

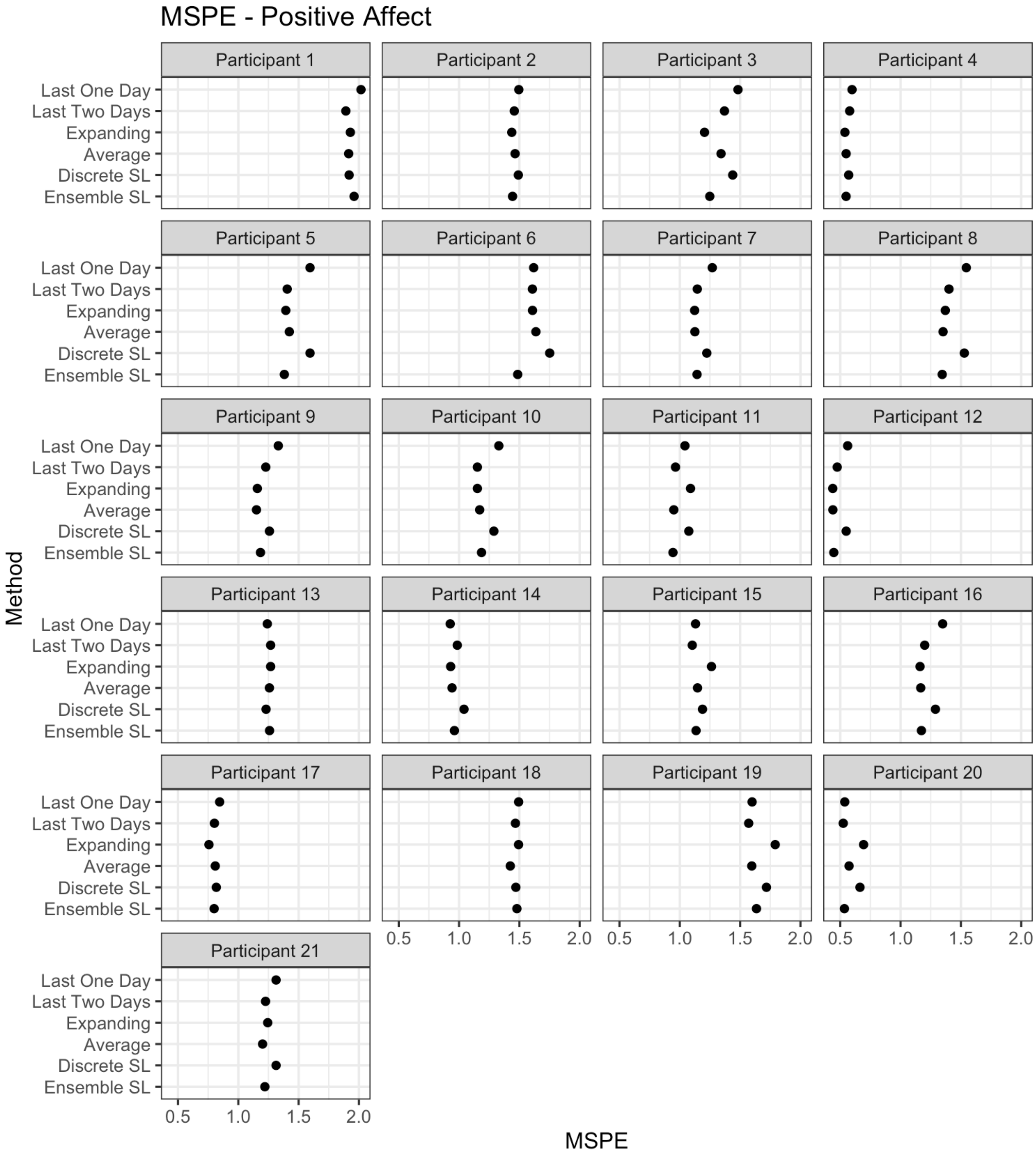

Figure S8

Time series as well as variance values for four example participants that are reported in the main manuscript.

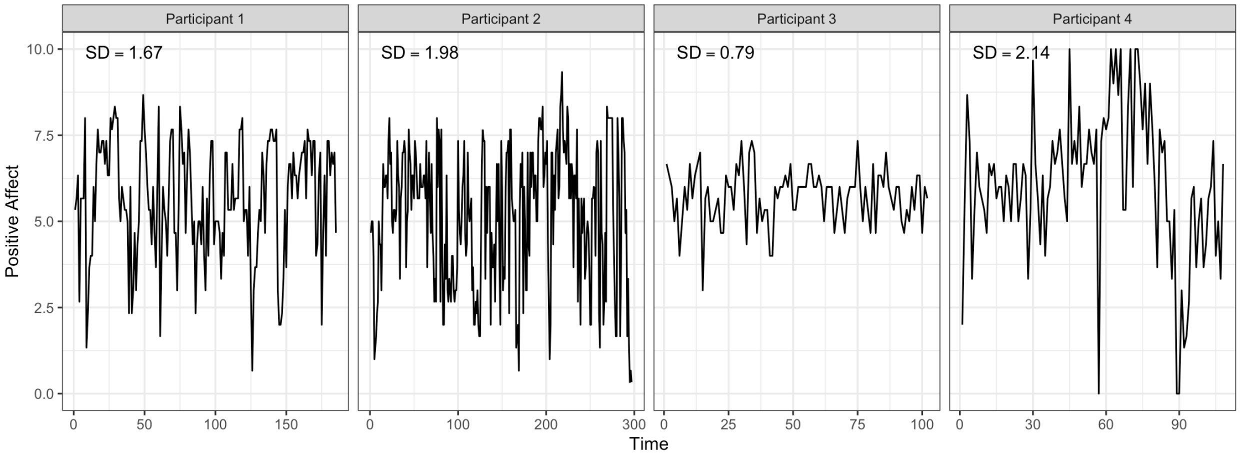

Figure S9

Distribution of the passive sensing feature of number of apps opened across different aggregation windows (1h, 3h, 6h, 9h, 12h, 24h) for a single participant. Top row: histograms showing frequency distributions. Bottom row: violin plots with individual data points. Data illustrate how aggregation window size affects the distribution and range of measured values for a single participant.

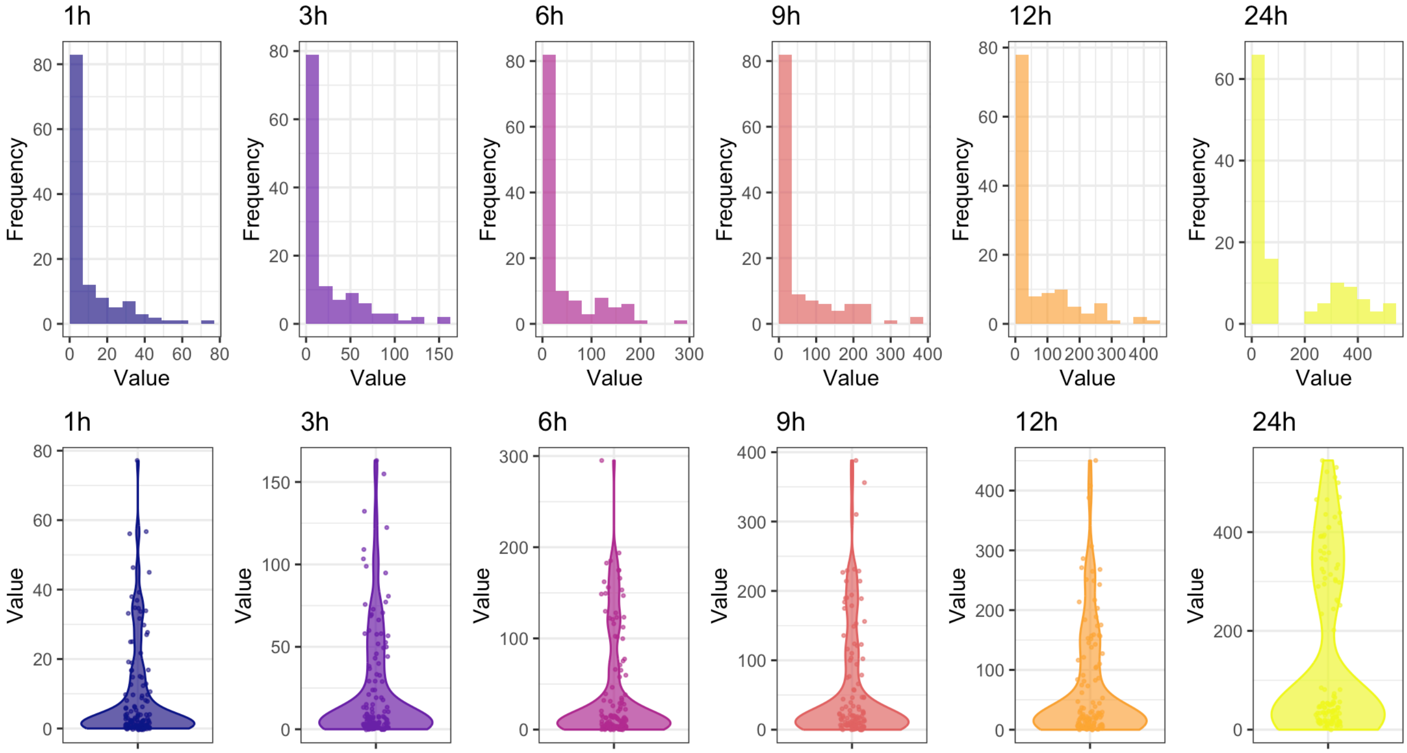

Figure S10

Distribution of the passive sensing feature of number of apps opened across different aggregation windows (1h, 3h, 6h, 9h, 12h, 24h) for a different participant than in Figure S9. Top row: histograms showing frequency distributions. Bottom row: violin plots with individual data points. Data illustrate how aggregation window size affects the distribution and range of measured values for a single participant.

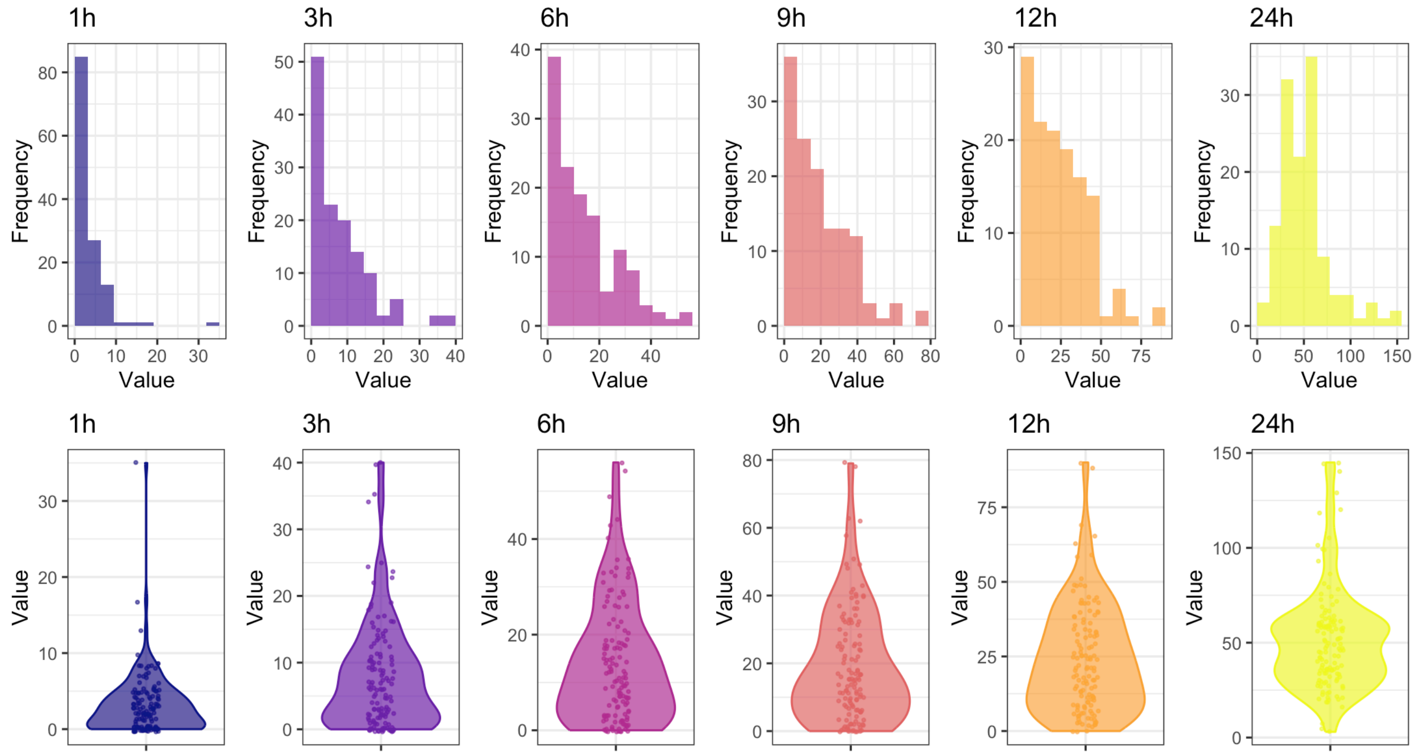

List of all covariates

All variables were aggregated over specific time windows (1–24 hours) before each experience sampling method (ESM) questionnaire. For details, see Langener et al. (2024).

Table S1

Summary of Digital Phenotyping Covariates by Category

Category	Number of Variables	Description
App Usage	29	Time spent on various app categories
Location/GPS	33	Time at different locations and mobility metrics
Social Sensing	4	Bluetooth/Wi-Fi connections as social proxies
Phone Calls	8	Call duration, frequency, and contact metrics
SMS Activity	3	Text messaging frequency and contacts
Environmental	2	Ambient light sensor data
Screen Activity	2	Screen on/off events
**Total**	**80**	**Unique passive smartphone measures**

Table S2

Complete List of 80 Passive Smartphone Features Used as Covariates

Variable	Type	Unit	Category
APP_USAGE_min	Continuous	Minutes	App Usage
APPS_OPENED_number	Count	Number	App Usage
COMMUNICATION_min	Continuous	Minutes	App Usage
PERSONALIZATION_min	Continuous	Minutes	App Usage
SOCIAL_min	Continuous	Minutes	App Usage
TOOLS_min	Continuous	Minutes	App Usage
BUSINESS_min	Continuous	Minutes	App Usage
MUSIC_AUDIO_min	Continuous	Minutes	App Usage
TRAVEL_LOCAL_min	Continuous	Minutes	App Usage
PHOTOGRAPHY_min	Continuous	Minutes	App Usage
PRODUCTIVITY_min	Continuous	Minutes	App Usage
com.whatsapp_min	Continuous	Minutes	App Usage
FINANCE_min	Continuous	Minutes	App Usage
SHOPPING_min	Continuous	Minutes	App Usage
NEWS_MAGAZINES_min	Continuous	Minutes	App Usage
HEALTH_FITNESS_min	Continuous	Minutes	App Usage
VIDEOPLAYERS_EDITORS_min	Continuous	Minutes	App Usage
STRATEGY_min	Continuous	Minutes	App Usage
WEATHER_min	Continuous	Minutes	App Usage
ENTERTAINMENT_min	Continuous	Minutes	App Usage
EDUCATION_min	Continuous	Minutes	App Usage
FOOD_DRINK_min	Continuous	Minutes	App Usage
LIFESTYLE_min	Continuous	Minutes	App Usage
PUZZLE_min	Continuous	Minutes	App Usage

Continued on next page

Table S2 – *Continued*

**Variable**	**Type**	**Unit**	**Category**
COMICS_min	Continuous	Minutes	App Usage
BOOKS_REFERENCE_min	Continuous	Minutes	App Usage
TRIVIA_min	Continuous	Minutes	App Usage
WORD_min	Continuous	Minutes	App Usage
MAPS_NAVIGATION_min	Continuous	Minutes	App Usage
Cluster_HOME_min	Continuous	Minutes	Location
TIME_STATIONARY_min	Continuous	Minutes	Location
UNIQUE_STAYPOINTS_number	Count	Number	Location
Cluster_1_min	Continuous	Minutes	Location
Cluster_2_min	Continuous	Minutes	Location
Cluster_3_min	Continuous	Minutes	Location
Cluster_4_min	Continuous	Minutes	Location
Cluster_5_min	Continuous	Minutes	Location
Cluster_6_min	Continuous	Minutes	Location
Cluster_7_min	Continuous	Minutes	Location
Cluster_8_min	Continuous	Minutes	Location
Cluster_9_min	Continuous	Minutes	Location
Cluster_10_min	Continuous	Minutes	Location
Cluster_11_min	Continuous	Minutes	Location
Cluster_12_min	Continuous	Minutes	Location
Cluster_13_min	Continuous	Minutes	Location
Cluster_14_min	Continuous	Minutes	Location
Cluster_15_min	Continuous	Minutes	Location
Cluster_16_min	Continuous	Minutes	Location
Cluster_17_min	Continuous	Minutes	Location
Cluster_18_min	Continuous	Minutes	Location
Cluster_19_min	Continuous	Minutes	Location
Cluster_20_min	Continuous	Minutes	Location
Cluster_21_min	Continuous	Minutes	Location
Cluster_22_min	Continuous	Minutes	Location
Cluster_23_min	Continuous	Minutes	Location
Cluster_24_min	Continuous	Minutes	Location
Cluster_25_min	Continuous	Minutes	Location
Cluster_26_min	Continuous	Minutes	Location
Cluster_27_min	Continuous	Minutes	Location
Cluster_28_min	Continuous	Minutes	Location
Cluster_29_min	Continuous	Minutes	Location
Cluster_30_min	Continuous	Minutes	Location
TOTAL_MACHASHES_number	Count	Number	Social Sensing
UNIQUE_MACHASHES_number	Count	Number	Social Sensing
BLUETOOTH_TOTAL_MACHASHES_number	Count	Number	Social Sensing

Continued on next page

Table S2 – *Continued*

**Variable**	**Type**	**Unit**	**Category**
BLUETOOTH_UNIQUE_MACHASHES_number	Count	Number	Social Sensing
CALL_TOTAL_min	Continuous	Minutes	Phone Calls
CALL_incoming_min	Continuous	Minutes	Phone Calls
CALL_outgoing_min	Continuous	Minutes	Phone Calls
MISSED_CALLS_number	Count	Number	Phone Calls
CALL_TOTAL_number	Count	Number	Phone Calls
CALL_incoming_number	Count	Number	Phone Calls
CALL_outgoing_number	Count	Number	Phone Calls
CALL_UNIQUE_CONTACTS_number	Count	Number	Phone Calls
SMS_received_number	Count	Number	SMS
SMS_sent_number	Count	Number	SMS
SMS_UNIQUE_CONTACTS_number	Count	Number	SMS
LIGHT_LUX_mean	Continuous	Lux	Environmental
LIGHT_LUX_std	Continuous	Lux	Environmental
SCREEN_onLocked_number	Count	Number	Screen Activity
SCREEN_onUnlocked_number	Count	Number	Screen Activity

References

Balliu, B., Douglas, C., Seok, D., Shenhav, L., Wu, Y., Chatzopoulou, D., ... Flint, J. (2024, February). Personalized mood prediction from patterns of behavior collected with smartphones. *npj Digital Medicine*, *7*(1), 49. Retrieved 2024-10-11, from https://www.nature.com/articles/s41746-024-01035-6 doi: 10.1038/s41746-024-01035-6

Langener, A. M., Stulp, G., Jacobson, N. C., Costanzo, A., Jagesar, R. R., Kas, M. J., & Bringmann, L. F. (2024, January). Its All About Timing: Exploring Different Temporal Resolutions for Analyzing Digital-Phenotyping Data. *Advances in Methods and Practices in Psychological Science*, *7*(1), 25152459231202677. Retrieved 2024-10-26, from http://journals.sagepub.com/doi/10.1177/25152459231202677 doi: 10.1177/25152459231202677

Makridakis, S., Spiliotis, E., & Assimakopoulos, V. (2020, January). The M4 Competition: 100,000 time series and 61 forecasting methods. *International Journal of Forecasting*, *36*(1), 54–74. Retrieved 2024-09-17, from https://www.sciencedirect.com/science/article/pii/S0169207019301128 doi: 10.1016/j.ijforecast.2019.04.014

Van Der Laan, M. J., Polley, E. C., & Hubbard, A. E. (2007, January). Super Learner. *Statistical Applications in Genetics and Molecular Biology*, *6*(1). Retrieved 2024-06-17, from https://www.degruyter.com/document/doi/10.2202/1544-6115.1309/html doi: 10.2202/1544-6115.1309
